# Turn-Off Fluorescent Sensor Based on 3-Aminophenylboronic Acid-Modified CdSe/ZnS Quantum Dots for Specific Detection of γ-Hexachlorocyclohexane (Lindane)

**DOI:** 10.3390/molecules31172989

**Published:** 2026-08-26

**Authors:** Dongdong Shi, Guiqin Yan

**Affiliations:** 1 School of Chemistry and Chemical Engineering, Shanxi Normal University, Taiyuan 030031, China; 118111005@stu.sxnu.edu.cn; 2 School of Life Sciences, Shanxi Normal University, Taiyuan 030031, China

**Keywords:** γ-hexachlorocyclohexane (γ-HCH), CdSe/ZnS-COOH, CdSe/ZnS-COOH@3-APBA, fluorescent probe

## Abstract

Herein, a highly selective and sensitive fluorescent sensing system is developed for the accurate quantitative determination of γ-Hexachlorocyclohexane (γ-HCH, Lindane). With carboxyl-functionalized CdSe/ZnS core–shell quantum dots (QDs) as the substrate, 3-Aminophenylboronic acid (3-APBA) is covalently conjugated onto the quantum dot surface via an amidation reaction. Systematic characterizations are used to confirm the successful fabrication of the CdSe/ZnS-COOH@3-APBA fluorescent probe, which exhibits excellent luminescence properties and superior colloidal dispersion stability. Under optimal experimental conditions, an increased γ-HCH concentration induces gradual attenuation of the probe fluorescence intensity. A favorable linear correlation is achieved within 10–140 nM, and the limit of detection is 2.62 nM. Mechanistic studies reveal that γ-HCH binds to 3-APBA on the probe surface via halogen bonding to form non-fluorescent ground-state association complexes, thereby triggering static quenching of the probe. This method can effectively distinguish α-, β-, and δ-hexachlorocyclohexane isomers. With the merits of simple operation, high sensitivity, excellent stability, and strong anti-interference capability, it provides a new strategy for the rapid screening and accurate quantification of γ-HCH residues in environmental and food matrices.

## 1. Introduction

Hexachlorocyclohexane (HCH) is a representative organochlorine persistent organic pollutant that exists as multiple isomers (α-, β-, γ-, and δ-HCH) [1,2]. Although the agricultural application of this class of pesticides has long been officially banned, all HCH isomers exhibit high chemical stability, pronounced toxicity, resistance to degradation, and strong bioaccumulation potential [3,4], and they also differ significantly in their toxicological effects and environmental mobility [5,6]. Among them, γ-HCH poses the most prominent carcinogenic and endocrine-disrupting risks [6]: long-term exposure to it can cause severe impairment to human hepatic and renal functions [7] and disrupt reproductive development [8], thereby posing a persistent threat to ecosystems and public health. Currently, residual HCH contamination in soil and water matrices and its illegal circulation remain issues in urgent need of regulation [7].

Conventional analytical techniques for HCH detection encompass bioanalytical assays [9,10], gas chromatography–mass spectrometry (GC-MS) [11,12], Surface-Enhanced Raman Scattering (SERS) [13], and electrochemical sensing platforms [14,15]; while these methods deliver high detection precision, they are universally constrained by inherent drawbacks: bulky analytical instrumentation, laborious sample pretreatment workflows, and elevated analytical costs. More critically, most conventional detection strategies fail to achieve specific recognition of individual HCH isomers and are susceptible to cross-signal interference among isomers, rendering them unable to satisfy the core requirements of on-site environmental monitoring: rapid response, operational simplicity, low cost, and high isomeric selectivity. Accordingly, the development of a facile, rapid, and highly sensitive fluorescent sensing system with excellent isomer discrimination capability for accurate qualitative and quantitative determination of γ-HCH in agricultural, food, and environmental matrices holds important theoretical value and practical application significance for ecological pollution source tracing, food safety regulation, and human health protection.

CdSe/ZnS core–shell QDs exhibit remarkable qualities, including tunable emission wavelength [16], high fluorescence quantum yield [17], outstanding photostability, uniform particle size, and facile surface functionalization [18], while also possessing favorable aqueous dispersibility and energy-level structures compatible with photoinduced electron transfer (PET) [19]. Therefore, they have been extensively exploited in trace fluorescent sensing of environmental pollutants [20], biological imaging [21], biochemical analysis [22], and other fields, serving as ideal nanomaterials for fabricating high-sensitivity and high-selectivity fluorescent nanoprobes [23].

**Scheme 1:**
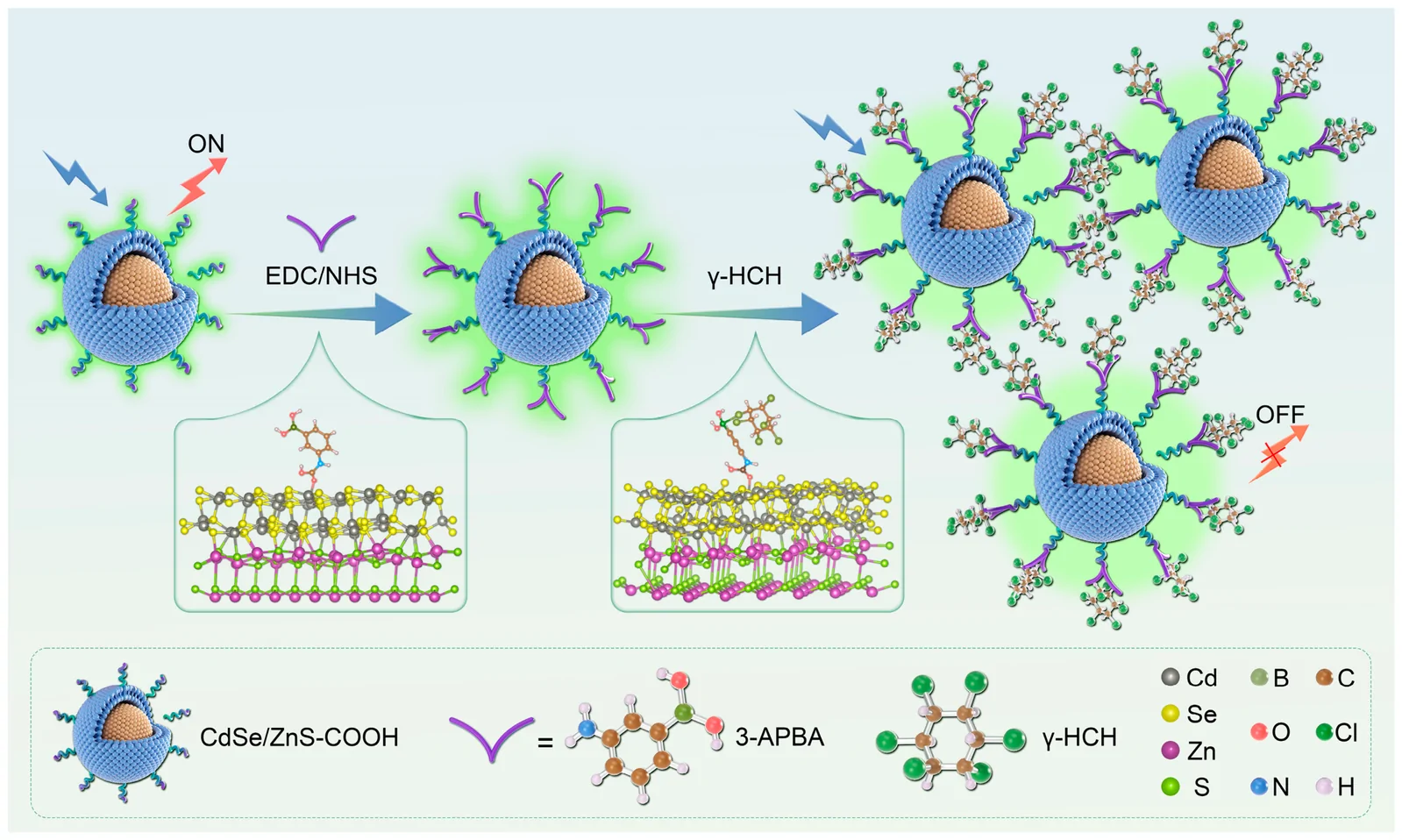
Schematic illustration of the CdSe/ZnS-COOH@3-APBA fluorescent probe for γ-HCH detection.

In view of the above advantages, we construct a 3-APBA-modified CdSe/ZnS-COOH core–shell QD fluorescent sensing system for the sensitive and selective determination of trace HCH, which achieves specific recognition of γ-HCH (Scheme 1). In this sensing system, weak halogen–Lewis coordination occurs between the boron atom of APBA and chlorine atoms of HCH, generating a non-fluorescent, thermodynamically stable ground-state complex, which deactivates luminescent centers. These two effects synergistically give rise to a dual-quenching mechanism characterized by the depletion of ground-state luminescent species and enhanced non-radiative relaxation of excited-state charge carriers. This recognition mechanism is fundamentally distinct from those of conventional quantum dot and phenylboronic acid-based pesticide-sensing systems. Current quantum dot sensors achieve recognition mainly via molecular imprinting [24], ion mediation [25,26], enzyme catalysis [27,28] and coordination binding [29]. Nevertheless, their underlying recognition mechanisms rely on common physicochemical interactions, including hydrogen bonds, van der Waals forces, hydrophobic interactions, electrostatic interactions, and electron transfer. Most reported boronic acid-functionalized fluorescent probes follow two recognition pathways: covalent complexation between boronic acid [30] and vicinal diols [25], and competitive displacement at polar functional group sites. Both mechanisms strongly depend on vicinal diol moieties and polar functional groups within analytes, and thus fail to recognize the non-polar organochlorine pollutant γ-HCH, which lacks hydroxyl and vicinal diol groups and only features carbon–hydrogen–chlorine skeletons. In contrast, this work develops a halogen bond-mediated recognition strategy to achieve the specific detection of γ-hexachlorocyclohexane.

In summary, the fluorescent sensing system fabricated by immobilizing 3-aminophenylboronic acid onto carboxylated CdSe/ZnS core–shell quantum dots (CdSe/ZnS-COOH@3-APBA) exhibits excellent detection sensitivity and specific recognition performance toward γ-HCH. Driven by this static quenching-dominated mechanism, the fluorescence signal of CdSe/ZnS quantum dots at 520 nm is attenuated in a regular manner with increasing γ-HCH concentration. The target analyte can be precisely quantified based on this concentration-dependent fluorescence quenching response. The sensing system delivers stable and reliable analytical performance for detection in real environmental and food samples, and addresses several drawbacks of conventional detection methods, including cumbersome sample pretreatment procedures, difficulty in distinguishing various hexachlorocyclohexane isomers, and detection signal interference originating from coexisting components in complex matrices. This work provides a novel and stable fluorescent sensing strategy for the rapid quantitative analysis of trace γ-hexachlorocyclohexane in environmental and food systems, and further broadens the application scope of CdSe/ZnS core–shell quantum dots in the detection of organochlorine pollutants.

## 2. Results and Discussion

### 2.1. Characterization

**Figure 1:**
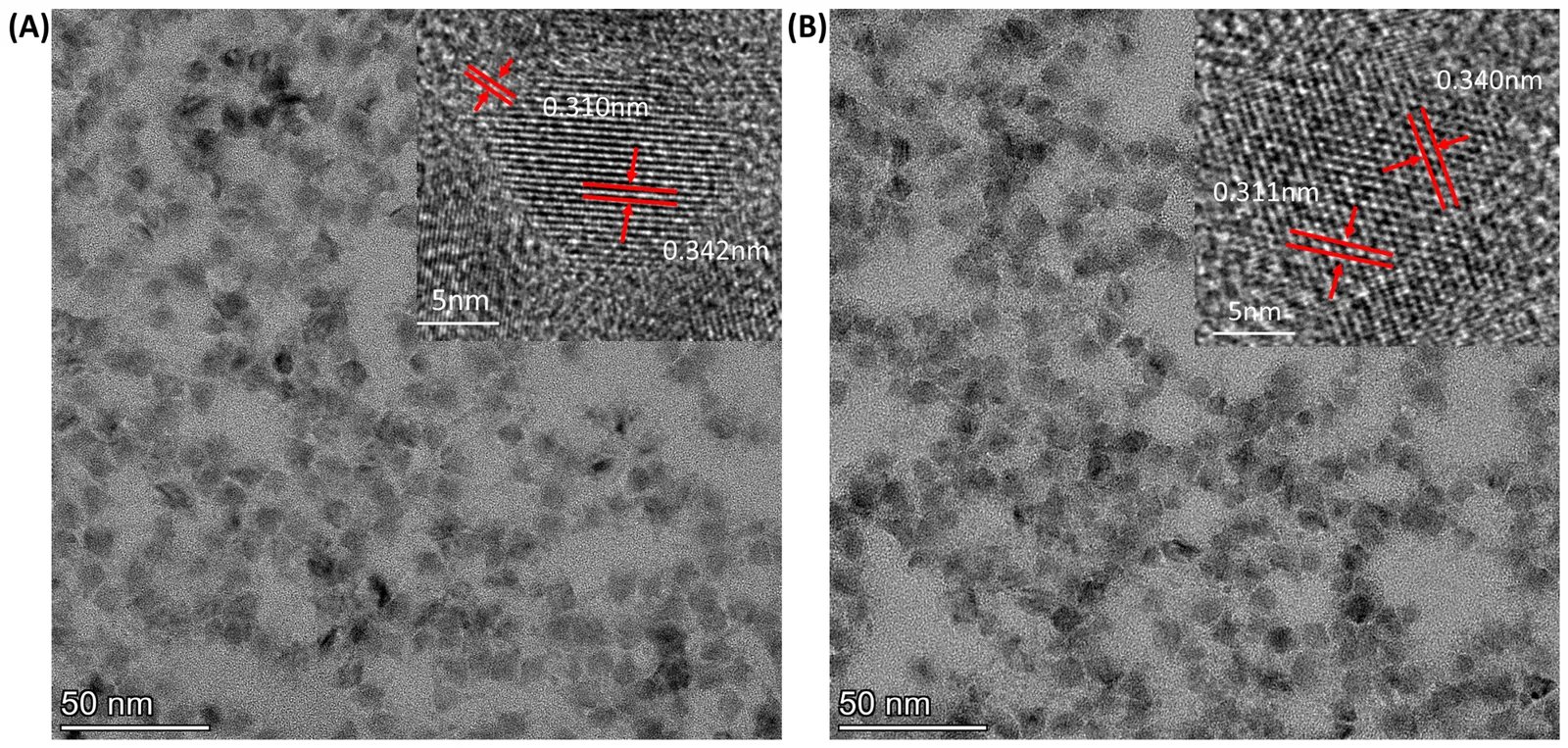
TEM and HRTEM characterization of CdSe/ZnS-COOH before and after modification. (**A**) TEM micrograph of pristine CdSe/ZnS-COOH, with the inset showing the corresponding HRTEM lattice fringes; (**B**) TEM micrograph of CdSe/ZnS-COOH@3-APBA, with HRTEM lattice fringes shown in the inset.

Transmission electron microscopy (TEM) micrographs show that CdSe/ZnS-COOH quantum dots exhibit near-spherical morphology and good monodispersity (Figure 1A), and statistical particle size analysis yields an average diameter of 8.04 ± 1.56 nm, with a main size distribution ranging from 7 to 10 nm (Figure S1) [31]. Well-resolved lattice fringes are clearly visible in the high-resolution transmission electron microscopy (HRTEM) image: core lattice spacings of 0.342 nm and 0.340 nm are indexed to the (111) plane of zinc-blende CdSe, while the shell lattice spacing of 0.310 nm and 0.311 nm agrees well with the theoretical value (0.311 nm) of the ZnS (111) plane [32]. After surface modification with 3-APBA, the average particle size, lattice spacing, and characteristic core–shell interfacial features remain nearly identical to those of the pristine QDs (Figure 1B); only surface functional group substitution takes place, and the internal crystal structure is not impaired by the modification process.

Steady-state fluorescence (SSF) measurements reveal that the pristine CdSe/ZnS-COOH QDs exhibit a strong green emission peak at 520 nm with a fluorescence intensity of 859 a.u. and a full width at half maximum (FWHM) of 40 nm (Figure S2). The Stokes shift between the 360 nm absorption peak and the 520 nm emission peak reaches 160 nm (Figure S2A), which effectively eliminates interference from excitation light and background fluorescence. The absolute fluorescence quantum yield of pristine CdSe/ZnS-COOH is determined to be 43.6%. After modification with 3-APBA, the emission position remains fixed at 520 nm (Figure S2B), while the fluorescence intensity decreases to 780 a.u. and the quantum yield stays at 38.2%. The emission band of the QDs exhibits slight spectral broadening, which is primarily ascribed to the formation of defect sites on the QD surface [33]. Nevertheless, no shifts are observed in the excitation peak, emission peak, or Stokes shift. This result demonstrates that 3-APBA only modifies the outermost surface layer of the QDs without disrupting the intrinsic photoluminescent structure of the CdSe core and merely leads to a slight drop in photoluminescence efficiency.

**Figure 2:**
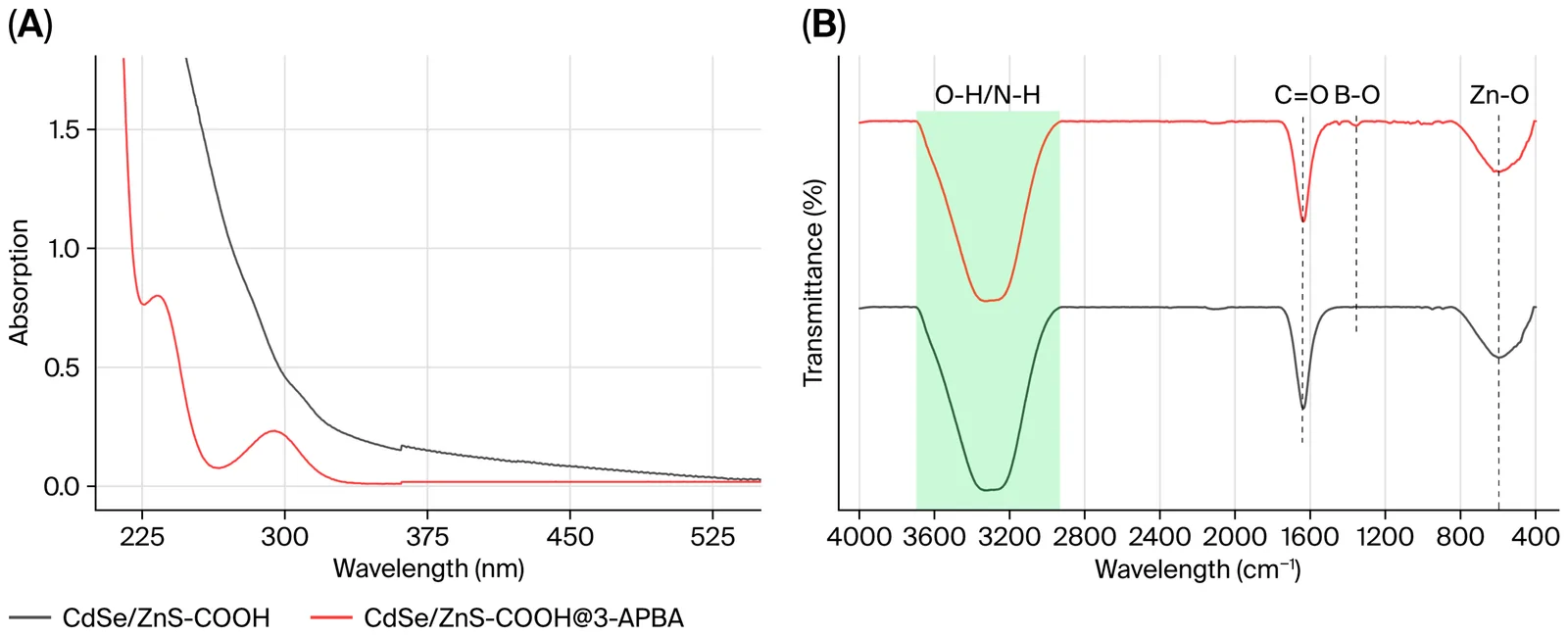
(**A**) UV-vis absorption spectra of CdSe/ZnS-COOH and CdSe/ZnS-COOH@3-APBA; (**B**) FT-IR spectra of CdSe/ZnS-COOH and CdSe/ZnS-COOH@3-APBA. (The green shaded area in (**B**) highlights the O-H/N-H absorption region, and dashed lines denote the positions of characteristic peaks including C=O, B-O and Zn-O).

Ultraviolet–visible (UV–vis) absorption spectra were recorded to verify successful modification with 3-APBA (Figure 2A). The pristine CdSe/ZnS-COOH QDs exhibit a characteristic excitonic absorption peak at 360 nm; after covalent grafting of 3-APBA, the excitonic peak of the modified QDs remains centered at 360 nm without any obvious shift. For the modified sample, two new absorption bands appear at 235 nm and 300 nm, which are attributed to the π→π* and *n*→π* electronic transitions originating from the benzene ring moiety of 3-APBA [34], directly confirming successful surface functionalization. No impurity-derived absorption peaks are observed throughout the measured spectral range, demonstrating that surface modification does not disrupt the intrinsic electronic energy levels or excitonic transition features of the QDs. The flat baseline recorded in the 375–540 nm region further confirms that the as-modified fluorescent probe retains good dispersion stability in aqueous media, with no signs of particle aggregation or sedimentation [35].

In Fourier transform infrared (FTIR) spectra (Figure 2B), a broad absorption band is observed in the 3000–3600 cm^−1^ region, which solely originates from the O–H stretching vibrations of free surface carboxyl groups for pristine CdSe/ZnS-COOH QDs; after 3-APBA modification, it remains nearly unchanged due to the overlap of N–H stretching vibrations of primary amine groups (–NH_2_) and O–H stretching vibrations of boronic acid moieties (–B(OH)_2_) from 3-APBA [35]. The intense absorption at 1600–1650 cm^−1^ is attributed to the C=O stretching vibrations of carboxyl and carboxylate groups. For the modified sample, a new characteristic absorption band appears at 1340–1360 cm^−1^, which corresponds to the B–O stretching vibrations of the boronic acid group in 3-APBA; no such signal is observed in pristine CdSe/ZnS-COOH QDs. The broad absorption at 600 cm^−1^ is assigned to Zn–O stretching vibrations arising from surface oxidation defects of the ZnS shell, and represents an intrinsic signal of the QDs [36]. Collectively, the differences in peak profiles and characteristic bands confirm the successful grafting of 3-APBA onto the surface of carboxylated CdSe/ZnS-COOH QDs. In summary, FTIR spectra preliminarily verify the grafting of 3-APBA onto carboxylated CdSe/ZnS-COOH quantum dots. However, weak, poorly resolved organic absorption bands and a flat baseline hinder reliable qualitative identification by FTIR alone. XPS measurements were therefore carried out to furnish complementary evidence for effective surface functionalization.

**Figure 3:**
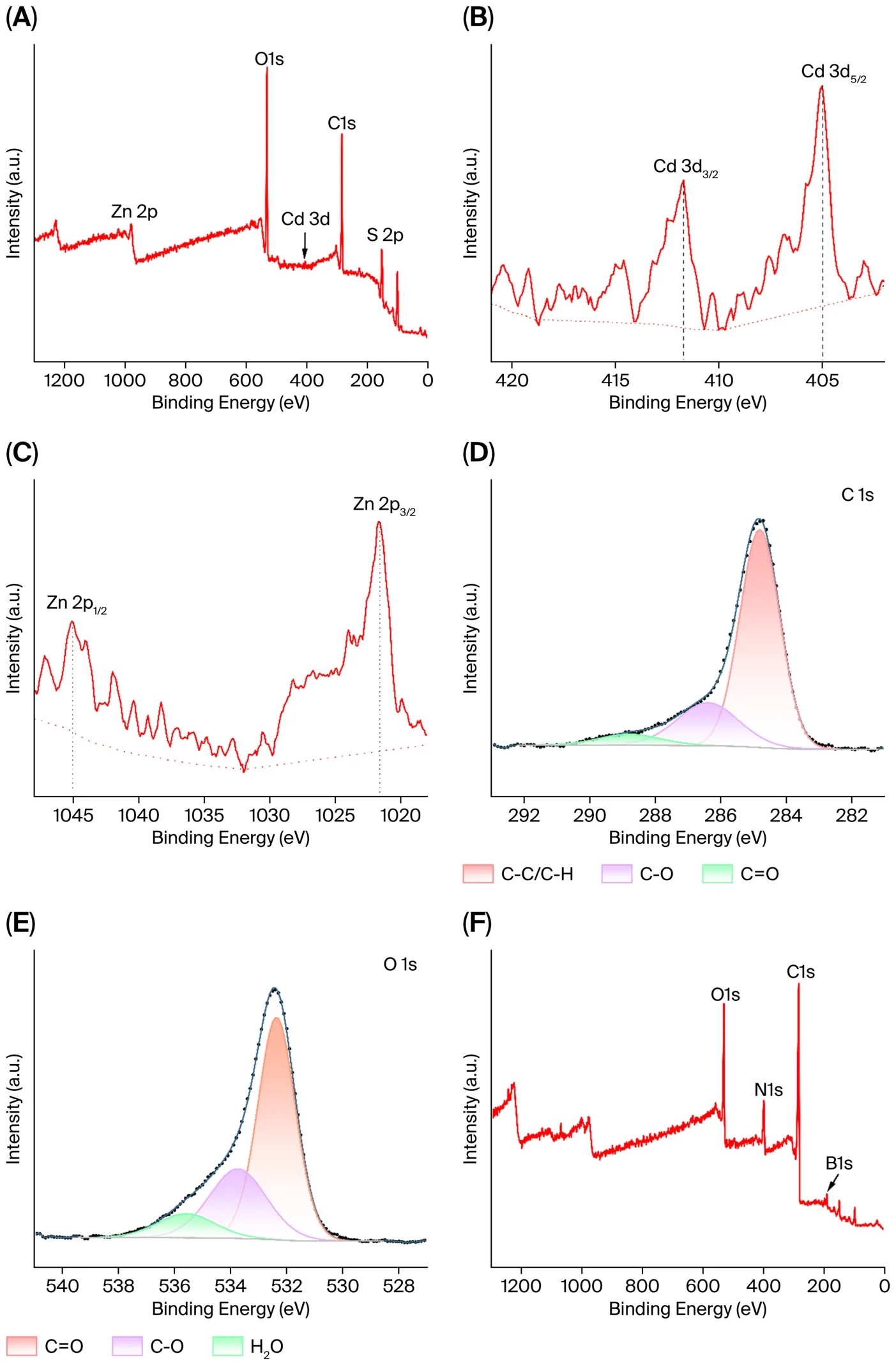

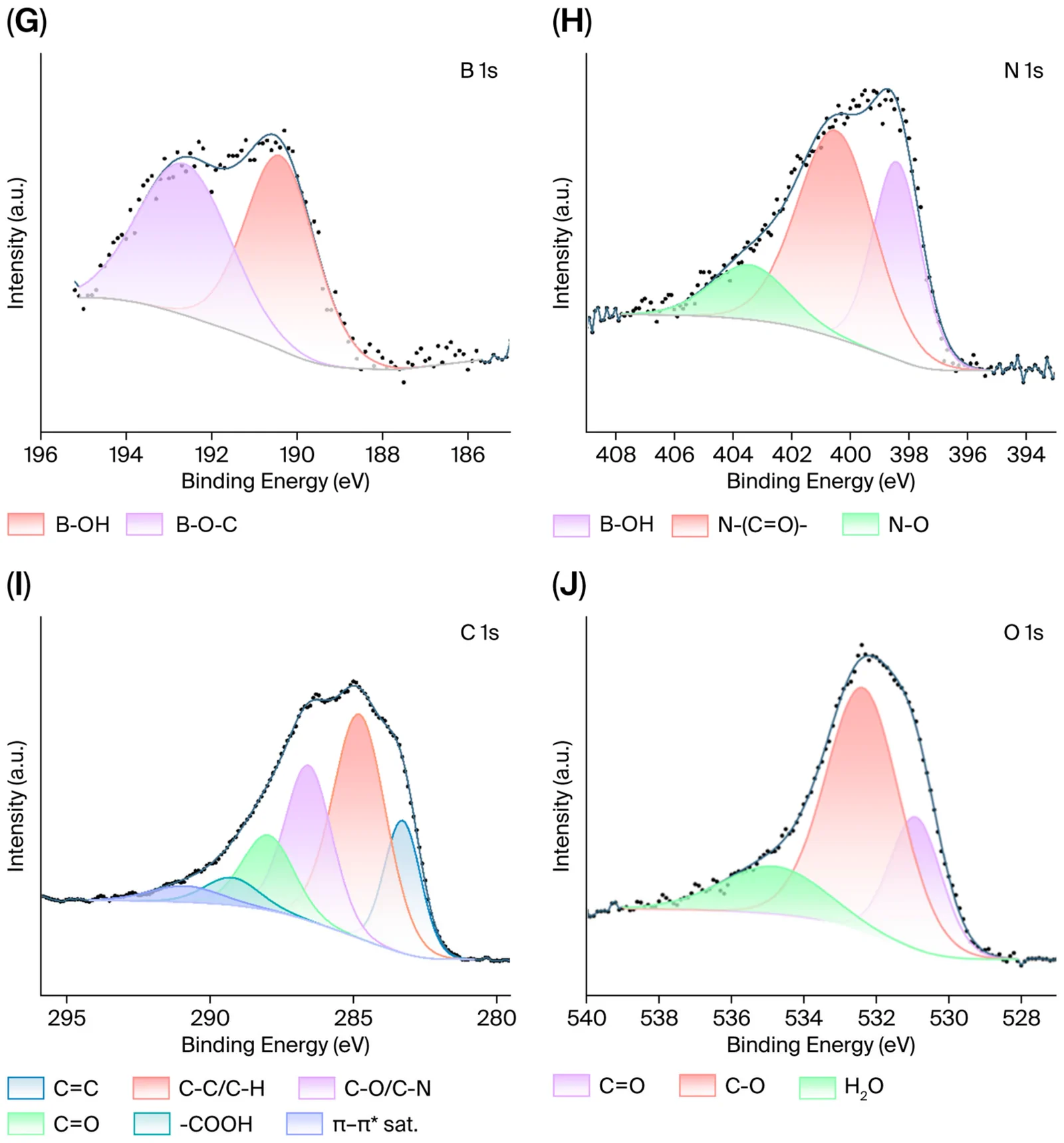
XPS of CdSe/ZnS-COOH and CdSe/ZnS-COOH@3-APBA. (**A**–**E**) Survey spectrum and high-resolution fitted spectra of Cd 3d, Zn 2p, C 1s, O 1s for CdSe/ZnS-COOH; (**F**–**J**) survey spectrum and high-resolution fitted spectra of B 1s, N 1s, C 1s, O 1s for CdSe/ZnS-COOH@3-APBA. (Vertical black dashed lines mark characteristic binding-energy positions. The red dotted lines correspond to the Shirley background for XPS spectral deconvolution. Black-blue solid lines denote fitted spectral curves, and black dots represent raw experimental XPS data).

X-ray photoelectron spectroscopy (XPS) was employed to characterize the as-synthesized CdSe/ZnS-COOH core–shell quantum dots (Figure 3). Fitting data of high-resolution spectra are summarized in Figure S3 and Tables S1–S3. The survey spectrum exhibits well-resolved characteristic signals of Zn 2p (0.79%), Cd 3d (0.25%), S 2p (5.06%), C 1s (61.01%), and O 1s (32.37%), with no detectable impurity-derived peaks (Figure 3A, Table S1) [37], and in high-resolution spectra, the Cd 3d spectrum exhibits two spin–orbit splitting doublets of Cd^2+^ at 405 eV and 412 eV (Figure 3B), while the Zn 2p spectrum shows characteristic Zn^2+^ doublets at 1022 eV and 1045 eV (Figure 3C). These results verify that the metal cationic species in both the CdSe core and ZnS shell exist in a stable divalent state with negligible oxidative impurities. Deconvolution of the C 1s spectrum yields three characteristic components at 284.80 eV (C–C/C–H), 286.39 eV (C–O), and 288.77 eV (free carboxyl C=O); in good agreement (Figure 3D), the O 1s spectrum is deconvoluted into two components at 533.74 eV (C–O) and 532.36 eV (free carboxyl C=O) (Figure 3E). The combined evidence from both spectra corroborates that abundant free carboxyl groups are present on the quantum dot surface, which can act as reactive sites for amide condensation with amino ligands and lay the foundation for subsequent 3-APBA functionalization.

For the as-modified CdSe/ZnS-COOH@3-APBA sample, the survey spectrum retains all the aforementioned characteristic peaks from inorganic species, C and O, while new N 1s (9.60%) and B 1s (8.35%) signals characteristic of 3-APBA are clearly identified, preliminarily validating successful ligand incorporation (Figure 3F, Table S1) [38,39]. The B 1s spectrum can be deconvoluted into two characteristic peaks at 190.39 eV (B-OH) and 192.64 eV (B-O-C) (Figure 3G), confirming that intact phenylboronic acid recognition sites are anchored on the QD surface. The high-resolution N 1s spectrum displays a characteristic amide N–C=O peak at 400.49 eV (Figure 3H), directly demonstrating the formation of covalent amide bonds via dehydration condensation between carboxyl and amino moieties [35]. Marked differences in the relative peak areas of deconvoluted C 1s components are observed before and after modification (Figure 3I). For pristine CdSe/ZnS-COOH QDs, the free C=O component at 288.8 eV accounts for a relatively high proportion. Upon amide condensation between carboxyl and amino groups, the corresponding free carboxyl peak in the modified sample shifts to 288.00 eV with a substantially reduced peak area; meanwhile, a new characteristic peak ascribed to C–N appears at 286.57 eV, accompanied by a concurrent increase in the peak areas of phenyl C=C and π–π* satellite peaks. Consistently, the peak area of free carboxyl C=O at 530.91 eV in the O 1s spectrum declines after modification (60.85% to 23.10%), and the dominant peak arises from the combined contributions of amide-conjugated C=O and boronic acid B–O components (Figure 3J). Collectively, the binding energy shifts and relative peak area variations in functional groups provide comprehensive chemical evidence that 3-APBA is covalently grafted onto the CdSe/ZnS QD surface via amide linkages.

In this work, CdSe/ZnS core–shell quantum dots serve as the fluorescent sensing platform. Benefiting from the pronounced quantum confinement effect, the CdSe core exhibits a characteristic excitonic absorption peak at 360 nm and emits stable green fluorescence at 520 nm. The outer ZnS shell effectively passivates dangling bonds and defect states on the quantum dot surface, decreases the non-radiative recombination probability of photogenerated electron–hole pairs, and thus enhances the dispersibility, optical stability, and structural integrity of the quantum dots. To impart specific recognition capability to the quantum dots, amide condensation takes place between surface carboxyl groups of the quantum dots and aromatic amino groups of 3-APBA molecules, mediated by the EDC/NHS activation system, forming stable covalent amide bonds and thus enabling successful immobilization of 3-APBA on the quantum dot surface. Multiple characterization results collectively confirm that 3-APBA is bound to the quantum dots through robust covalent coupling rather than simple physical adsorption. Furthermore, the modification process does not compromise the intrinsic optical properties of the quantum dots, while the boronic acid groups and benzene ring structures of 3-APBA, which act as specific recognition sites for HCH, remain well-preserved.

### 2.2. Feasibility Evaluation of Detection

To verify the feasibility of the as-fabricated fluorescent probe for γ-HCH detection, comparative analysis of fluorescence spectra was conducted across different sample groups (Figure S4A). As illustrated in the figure, pristine CdSe/ZnS-COOH QDs exhibit excellent fluorescence emission performance. After covalent grafting of 3-APBA to construct the sensing probe, the fluorescence intensity undergoes a slight decline, which can be ascribed to the introduction of surface defects by the aromatic 3-APBA ligands that aggravate non-radiative carrier recombination [40]. Upon addition of γ-HCH into the modified probe system, pronounced fluorescence quenching is observed, with the quenching efficiency reaching up to 50%. However, complete fluorescence quenching cannot be achieved, which may be attributed to the limited quantity of effective binding sites, steric hindrance, molecular conformation and molecular dipole properties [41]. The control experiment reveals that no noticeable fluctuation in fluorescence intensity occurs when γ-HCH is directly incubated with bare CdSe/ZnS-COOH, verifying that the target analyte cannot directly quench the intrinsic fluorescence of the quantum dots.

We also selected four aromatic ligands free of boronic acid groups, namely aniline, benzoic acid, 4-mercaptobenzoic acid, and phenylalanine, and constructed multiple control quantum dot systems via covalent bonds and coordination interactions to explore their response behaviors toward γ-HCH. Upon the addition of γ-HCH, no evident fluorescence quenching occurred in the four control systems, whereas CdSe/ZnS-COOH@3-APBA exhibited prominent fluorescence quenching (Figure S4B). These preliminary results suggest that the sensing response of the probe toward γ-HCH is likely governed by boronic acid groups, rather than being mediated by aromatic skeletons or carboxyl, amino, or thiol groups.

These results collectively confirm that the as-developed probe enables targeted quantitative detection of γ-HCH, fully validating the favorable feasibility of the proposed detection strategy.

### 2.3. Mechanism of Fluorescence Quenching

**Figure 4:**
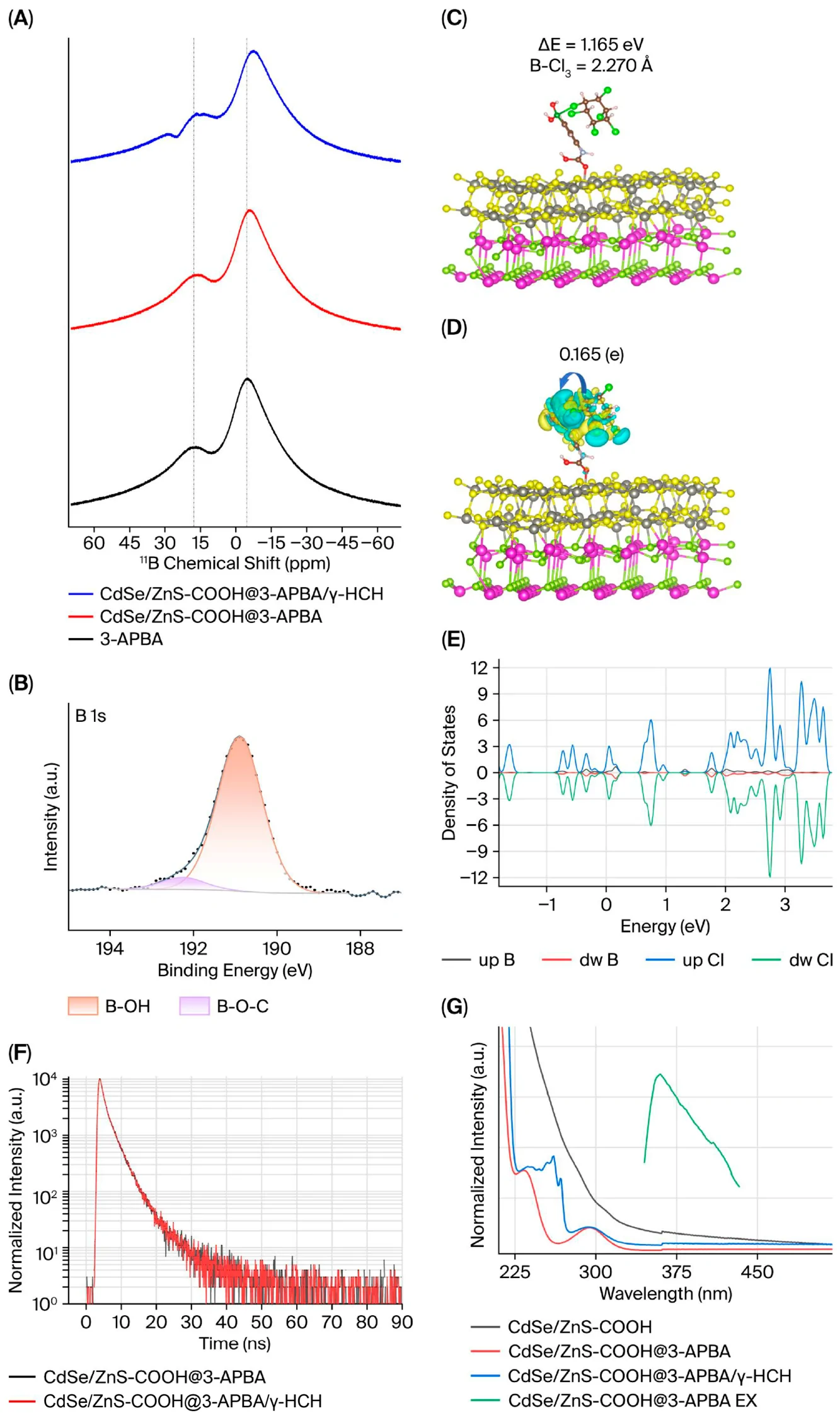
Spectroscopic characterizations and theoretical calculations revealing the interaction and fluorescence quenching mechanism between γ-HCH and the CdSe/ZnS-COOH@3-APBA probe. (**A**) ^11^B NMR spectra of free 3-APBA, CdSe/ZnS-COOH@3-APBA probe, and probe after incubation with γ-HCH; (**B**) high-resolution B 1s spectrum of the probe after interaction with γ-HCH (Black-blue solid lines denote fitted spectral curves, and black dots represent raw experimental XPS data); (**C**) optimized geometric adsorption configuration of γ-HCH on the probe surface obtained by DFT calculations; (**D**) charge density difference distribution (blue: charge accumulation, cyan: charge depletion); (**E**) PDOS curves of B and Cl atoms; (**F**) TRFL spectra of the probe before and after the addition of γ-HCH; (**G**) UV–vis absorption and fluorescence excitation spectra of different samples.

Upon the introduction of γ-hexachlorocyclohexane, significant fluorescence quenching of the CdSe/ZnS-COOH@3-APBA probe was observed. To elucidate the underlying recognition mechanism, the intermolecular interaction between the probe and γ-HCH was systematically investigated via ^11^B NMR and XPS measurements. The ^11^B NMR spectra show that free 3-APBA and CdSe/ZnS-COOH@3-APBA exhibit nearly identical boron resonance profiles, with only a slight upfield shift in the main peak. After adding γ-HCH, the main boron peak shifts further upfield, accompanied by obvious distortion of the low-field shoulder peak (Figure 4A). This demonstrates that the chlorine atoms of γ-HCH act as halogen bond donors and undergo Lewis acid–base interactions with the electron-deficient boron centers of 3-APBA, thereby altering the local chemical microenvironment of boron.

XPS tests further corroborate this interaction. After incubation with γ-HCH, characteristic Cl signals can be observed in the survey spectrum (Figure S5A), and the corresponding high-resolution Cl 2p spectrum is provided in the Supporting Information (Figure S5B). High-resolution B 1s spectra display an overall shift toward lower binding energy (Figure 4B). Meanwhile, the relative proportions of the two deconvoluted subpeaks at 190.39 eV (B–OH) and 192.64 eV (B–O–C) change considerably, with a remarkable increase in the B–OH component. This variation indicates that the formation of B–Cl halogen bonding weakens the boron–oxygen cross-linking structure on the probe surface, promoting the dissociation of B–O–C species and the regeneration of monomeric B–OH groups. Combined with the overall lower binding energy shift, these spectral changes confirm that halogen bonding induces charge transfer from Cl to B and increases the electron density of boron centers. Collectively, the ^11^B NMR and XPS results mutually verify that stable intermolecular complexes are formed between the probe and γ-HCH via B–Cl halogen bonding interactions.

To elucidate the underlying interaction mechanism, density-functional theory (DFT) calculations were performed to investigate the interfacial interaction and electron transfer behavior between CdSe/ZnS-COOH@3-APBA and γ-HCH at the atomic scale. As shown in Figure 4C, the optimized adsorption configuration reveals that γ-HCH is stably anchored on the probe surface via Lewis acid–base coordination interaction between the electron-deficient boron center of the APBA moiety and the chlorine atoms of γ-HCH [42], with a B–Cl interatomic distance of 2.270 Å. The absolute value of adsorption energy (ΔE) of the complex is calculated to be 1.165 eV, and such a large adsorption magnitude verifies the favorable thermodynamic stability of the formed structure. Figure 4D presents the charge density difference map with an isosurface value of 0.0002 e/Å^3^, focusing on the interfacial binding region between the probe and γ-HCH. Distinct electron accumulation and depletion regions are identified in the B–Cl interaction domain, with significant overlap and interpenetration of electron clouds, providing intuitive evidence for remarkable charge redistribution at the interface. Quantitative Bader charge analysis indicates that γ-HCH transfers a net charge of 0.165 e to the probe, confirming effective orbital hybridization between B and Cl atoms and the formation of an efficient interfacial electron transfer pathway. To eliminate intensity bias caused by differences in atomic number, peak normalization was applied to the partial density of state (PDOS) profiles (Figure 4E), and the normalized results show that the characteristic peaks of B and Cl orbitals are highly overlapped in the conduction band range of 0–2 eV with well-matched energy levels, further validating the orbital hybridization effect from the perspective of electronic energy levels. The above theoretical results indicate that intermolecular association can effectively perturb the electron cloud distribution and orbital energy levels of the aromatic ring in 3-APBA, reconstruct the electronic structure of the system, and eventually form non-emissive ground-state-associated complexes. This reveals the intrinsic mechanism of fluorescence quenching when the probe recognizes γ-hexachlorocyclohexane from the microscopic electronic perspective.

Generally, fluorescence quenching mechanisms include static quenching, dynamic quenching, fluorescence resonance energy transfer (FRET), photoinduced electron transfer (PET), and the inner filter effect (IFE). TRFL was adopted to characterize the excited-state dynamics of the system (Figure 4F), and the fluorescence decay curves were fitted and analyzed using a bi-exponential kinetic model (Table 1). For the pure probe system, two fluorescence lifetime components were obtained—τ_1_ = 1.0993 ns and τ_2_ = 3.5757 ns—with corresponding fractional contributions of 42.17% and 57.83%, and a goodness-of-fit χ^2^ = 1.421. After introducing γ-HCH, only slight fluctuations in lifetime parameters were observed—τ_1_ = 1.0630 ns, τ_2_ = 3.5103 ns—and the fractional contributions changed to 40.14% and 59.86%, with χ^2^ = 1.435. The goodness-of-fit values of the two samples are within the credible range, verifying that the bi-exponential model can effectively describe the fluorescence decay kinetics of the probe. No obvious regular attenuation of each lifetime component or significant variation in fractional contributions was observed after adding the target analyte. As an intrinsic parameter characterizing excited-state evolution, dynamic quenching induced by excited-state collisions is generally accompanied by a reduction in fluorescence lifetime. The nearly constant fluorescence lifetimes in this system indicate that the excited-state relaxation of free probes is not disturbed by intermolecular collisions, thereby excluding the dynamic quenching mechanism.

Combined with the results of UV–vis absorption spectroscopy, fluorescence kinetics, and DFT theoretical calculations, the fluorescence quenching mechanism triggered by γ-HCH was further explored. UV–vis absorption spectroscopy enables direct visualization of the evolutionary features of molecular ground-state electronic structures, acting as a critical criterion for identifying the presence of ground-state intermolecular interactions within the system. Compared with the pure probe system (Figure 4G), the addition of γ-HCH induces remarkable deformation and a moderate red shift in the probe’s characteristic absorption profile at 235 nm, whereas the absorption signals at 300 nm and 360 nm remain nearly invariant. The π→π* transition absorption band centered at 235 nm displays prominent spectral alterations, and these deviations cannot be attributed to simple superposition of the absorption signals originating from the probe and analyte, which solidly verifies that stable ground-state complexes can form between the probe and γ-HCH via intermolecular halogen bonding. Further analysis reveals that intermolecular B-Cl halogen bonding can markedly perturb the electron cloud distribution and orbital energy-level configuration of the 3-APBA aromatic ring, trigger electronic structure reconstruction of the system, and generate new electronic transition features. This manifests macroscopically as anomalous variations in the absorption profile at 235 nm. Such electronic structure reconstruction modulates the photophysical properties of the associated adducts, and the resulting ground-state complexes are non-fluorescent. In combination with fluorescence kinetic measurements, the fluorescence lifetimes of the system exhibit no appreciable decay. Meanwhile, γ-HCH shows no characteristic absorption within the emission window of the probe, failing to satisfy the prerequisite for FRET. Accordingly, PET and FRET quenching pathways can be ruled out. DFT theoretical calculations further corroborate the interfacial electron redistribution and orbital coupling characteristics, establishing a complete evidence chain covering macroscopic spectral observations and microscopic electronic structures. Comprehensive analysis of macroscopic spectroscopic characterizations and microscopic electronic structures confirms that the sensing system follows a static fluorescence quenching mechanism, and the decline in fluorescence intensity stems from the generation of non-fluorescent ground-state complexes.

On this basis, the interference originating from the inner filter effect was further excluded in this work (Figure 4G). CdSe/ZnS-COOH quantum dots exhibit an excitonic absorption shoulder at 360 nm, and the fluorescence excitation maximum of the modified probe is also located at 360 nm. The overlap between the absorption shoulder and excitation maximum represents an inherent photophysical property of fluorophores, indicating that electronic transitions at this wavelength can efficiently generate fluorescence. Such intrinsic spectral matching is a common feature and does not indicate the occurrence of the inner filter effect. Meanwhile, the UV absorption of γ-HCH is mainly concentrated in the short-wavelength region below 200 nm, and virtually no absorption is detected at 360 nm. The overall absorbance of samples at 360 nm remains low, and no obvious absorbance enhancement emerges after adding γ-HCH. These observations fully verify that fluorescence quenching primarily arises from the specific interaction between the probe and γ-HCH to form non-fluorescent ground-state complexes, rather than being caused by the inner filter effect.

In summary, the fluorescence quenching behavior of the system conforms to the static quenching mechanism: probe molecules can bind γ-HCH in the ground state to form non-fluorescent ground-state complexes, which ultimately induces fluorescence quenching.

### 2.4. Optimization of Detection Conditions

**Figure 5:**
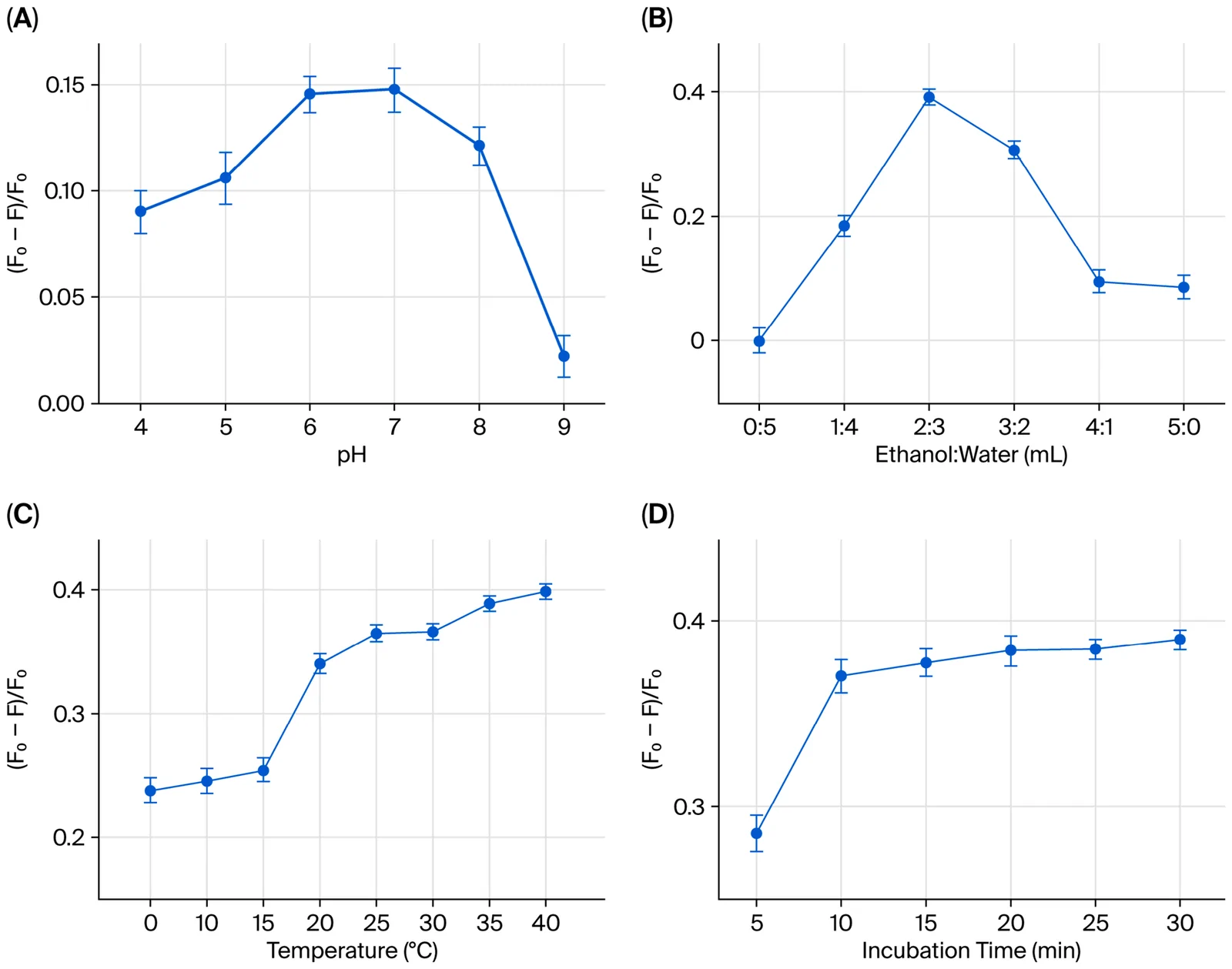
Parameter optimization of the constructed sensing system. Influence of (**A**) pH value; (**B**) ethanol–water volume ratio; (**C**) reaction temperature; (**D**) incubation time.

To optimize the sensing performance of the HCH detection system, the effects of pH value, ethanol-to-water volume ratio, reaction temperature, and incubation time on the detection response were systematically investigated. The results show that the probe achieves a relatively high fluorescence quenching efficiency under neutral to weakly alkaline conditions (pH 6–8) (Figure 5A). The solution pH can modulate the protonation state and spatial geometry of the boronic acid moieties in 3-APBA, simultaneously governing the anti-interference capacity against cis-diols and the interaction strength between boron sites and γ-HCH. Under weakly acidic conditions, boronic acid groups predominantly adopt a neutral sp^2^ planar geometry [43]. The boron center retains moderate electron deficiency, which prevents complexation with cis-diol impurities and enables the formation of stable weak intermolecular interactions with chlorine atoms of γ-HCH, eliciting distinct fluorescence quenching. At a strongly acidic pH, excess protons passivate the vacant orbitals of boron and weaken the recognition performance. In alkaline environments, boronic acid undergoes deprotonation to form sp^3^-hybridized tetrahedral anions [43]. These anions tend to bind polyhydroxy impurities and cause interference; additionally, their negative charges generate electrostatic repulsion toward γ-HCH, markedly lowering the detection sensitivity. Accordingly, pH = 6 serves as the optimal condition for striking a balance between preventing matrix interference and the reliable recognition of γ-HCH [44,45].

The solvent polarity and solvent type (protic/aprotic solvents) synergistically regulate the luminescence performance of the probe through solute–solvent interactions [46]. In the ethanol–water mixed medium, γ-hexachlorocyclohexane can be uniformly dispersed at an ethanol/water volume ratio of 2:3, and the probe exhibits favorable optical stability (Figure 5B). Therefore, this solvent ratio was adopted for subsequent measurements. The temperature gradient optimization results reveal that the fluorescence quenching efficiency induced by γ-HCH gradually increases with rising system temperature (Figure 5C); in view of the practical requirements for on-site rapid detection, room temperature (25 °C) was selected as the optimal reaction temperature. After the addition of γ-HCH, the relative fluorescence quenching efficiency remains nearly constant with prolonged incubation time (Figure 5D). The binding equilibrium of the system is reached after 10 min of incubation, and further extension of the reaction time causes no significant fluctuation in the quenching response signal [47]. Based on the above optimization results, this work provides a solid experimental basis for the construction of a highly sensitive and target-specific fluorescent platform for γ-HCH detection.

### 2.5. Sensitivity Analysis

**Figure 6:**
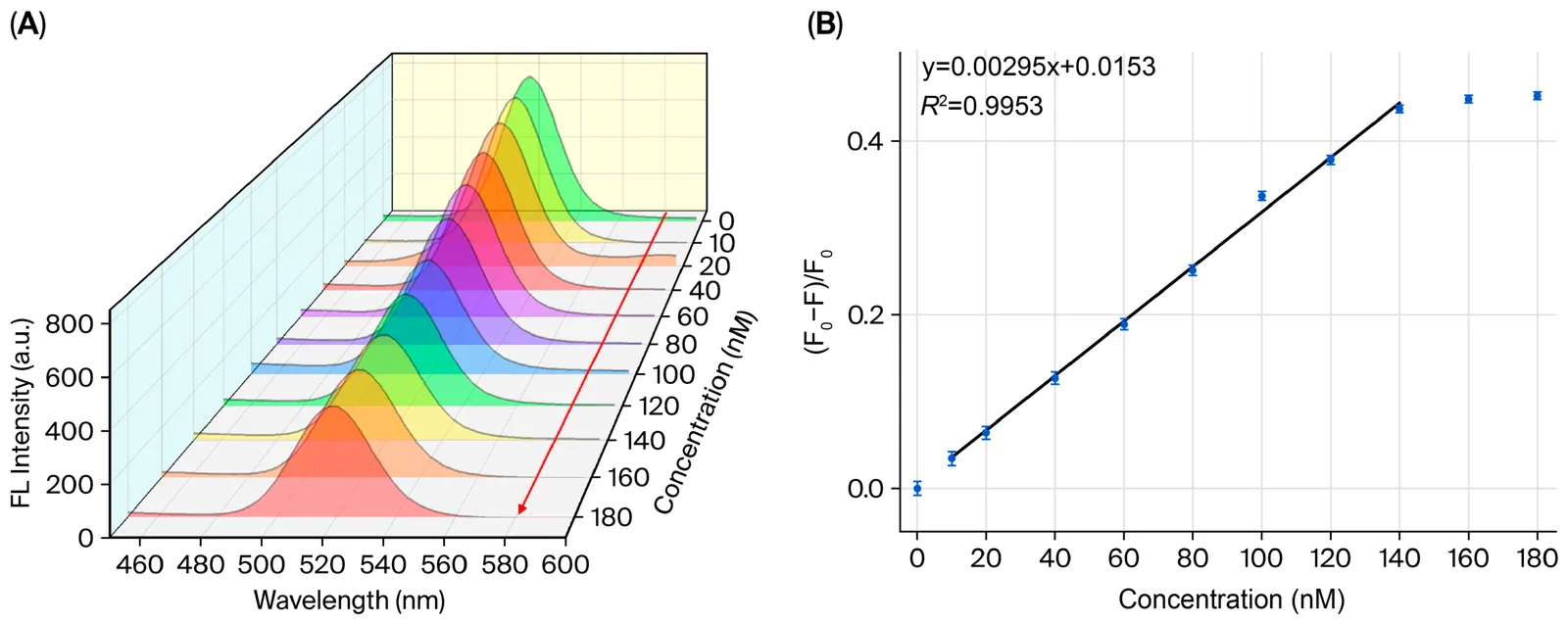
(**A**) Fluorescence emission spectra of CdSe/ZnS-COOH@3-APBA probe at various γ-HCH concentrations (0, 10, 20, 40, 60, 80, 100, 120, 140, 160 and 180 nM), The red arrow indicates the direction of fluorescence quenching with increasing γ-HCH concentration; (**B**) calibration curve of the sensor for γ-HCH at concentrations from 10 to 140 nM.

The fluorescence response of the probe toward γ-HCH standard solutions at concentrations ranging from 0 to 180 nM was examined under the optimal detection conditions (Figure 6A). The fluorescence spectra show that the probe fluorescence is quenched in a concentration-dependent manner with increasing γ-HCH concentration, and a favorable linear correlation between fluorescence quenching efficiency and target concentration is achieved in the range of 10–140 nM (Figure 6B), and all statistical inferences derived from the linear fitting are reliable (Figure S6). The linear regression equation was fitted as *y*= 0.00295*x* + 0.0153 (*R*^2^ = 0.9953), demonstrating excellent linearity. The limit of detection (LOD) was calculated based on the classical formula LOD = 3σ/s. Twelve parallel measurements of blank buffer solution were performed to obtain the standard deviation of blank signals (σ = 0.00258). Combined with the slope of the calibration curve (s = 0.00295), the LOD was determined to be 2.62 nM; this is lower than the maximum residue limits of γ-HCH in food and aquatic environments specified in the national standard, confirming that the proposed method meets the requirements for trace γ-HCH residue detection in environmental and food samples.

### 2.6. Specificity, Anti-Interference Performance and Stability

**Figure 7:**
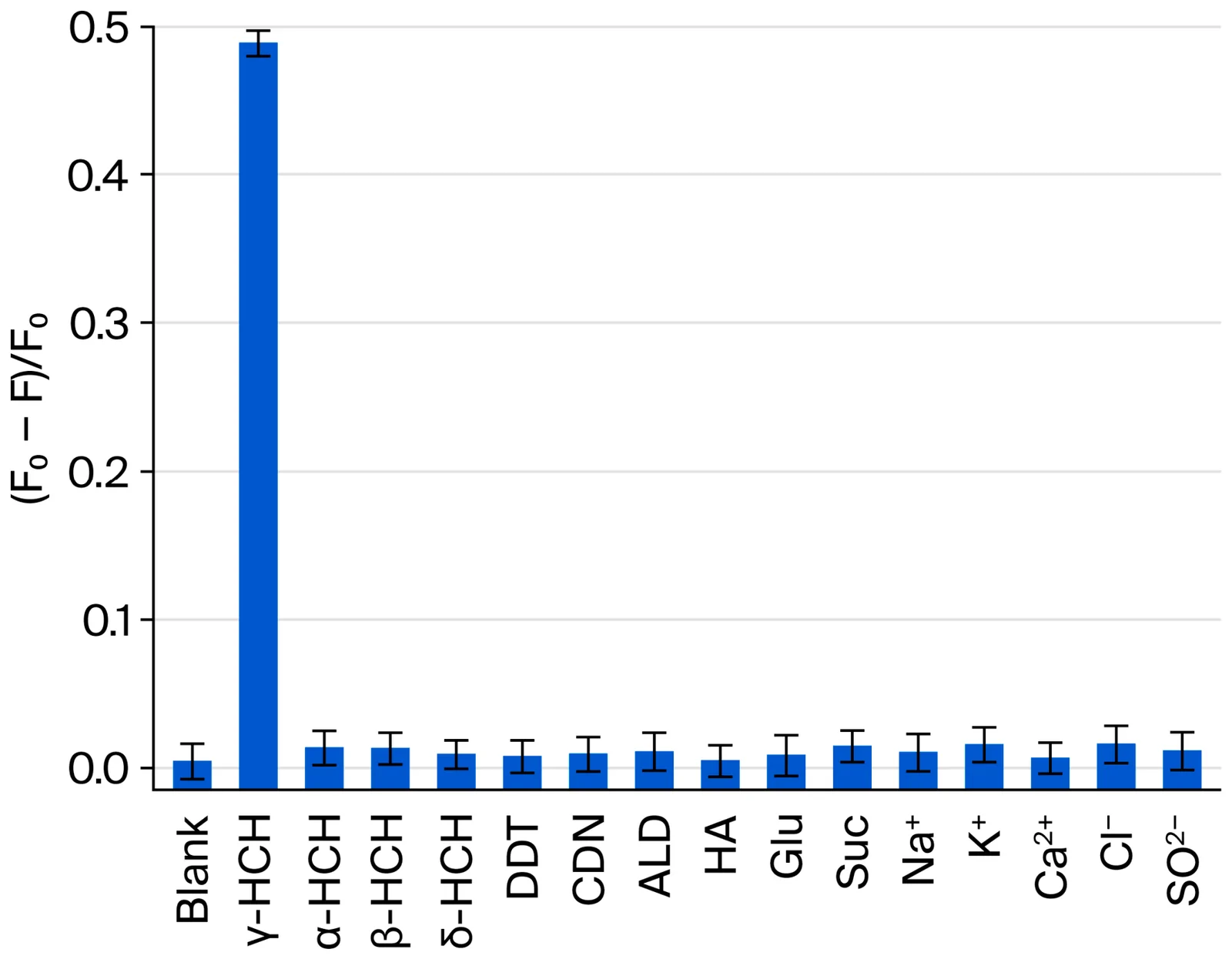
The specificity of the proposed probe to γ-HCH and other interfering substances.

To evaluate the target recognition specificity of the as-fabricated CdSe/ZnS-COOH@3-APBA probe toward γ-HCH, comparative experiments were performed using a series of structural analogs and common interfering substances, including its isomers (α-HCH, β-HCH, and δ-HCH), glucose, sucrose, and ubiquitous environmental cations /anions (Na^+^, K^+^, Ca^2+^, Cl^−^, and SO_4_^2−^) (Figure 7).

Their effects on probe fluorescence intensity were tested under identical concentration conditions, and only the target analyte γ-HCH induced significant fluorescence quenching, while all other tested substances exerted negligible influence. The distinct spatial configurations of HCH isomers directly modulate their interfacial binding strength with the probe [48,49]. Cyclohexane derivatives adopt two dominant conformations, stable chair and flexible twist-boat, the latter of which exhibits optimal thermodynamic stability in flexible skeletons [41]. Chlorine substitution patterns of hexachlorocyclohexane isomers modulate skeletal geometry, dipole moment and molecular electrostatic potential. Electrostatic potential, the molar Kerr constant, and the dipole moment enable the identification of liquid-phase-dominant conformations and the evaluation of B-Cl halogen bond strength. β-HCH adopts a highly symmetric all-equatorial chair conformation with a dipole moment of 0 D, α-HCH exhibits dynamic coexistence of chair and twist-boat conformations and δ-HCH exists as a distorted chair conformation; the latter two both possess inadequate dipole and polarization properties. In contrast, γ-HCH has a measured dipole moment of 2.65 D and a molar Kerr constant of −37.6 × 10^−12^ [41], with the twist-boat conformation predominating. This conformation breaks molecular symmetry, generates a favorable dipole moment and accumulates negative electrostatic potential around chlorine atoms. Owing to its excellent electronic polarizability, γ-HCH efficiently forms B-Cl halogen bonds with the modified probe. Molecular docking simulations were performed via HDOCKlite v1.1 [34]; PyMOL 2.5.3 was utilized for visual analysis of the optimal binding conformation to elucidate the molecular interaction mechanism between 3-aminophenylboronic acid and γ-HCH (Figure S7A). DFT adsorption energy calculations reveal that the probe–γ-HCH interaction yields the strongest adsorption energy (Δ*E* = −1.165 eV), whose absolute value is markedly higher than those of α-HCH, δ-HCH, andβ-HCH (Table S4) [50]. Fluorescence measurements show that only γ-HCH generates pronounced and stable fluorescence quenching after 10 min of incubation (Figure S7B), whereas the other three isomers exhibit only faint fluorescence attenuation over 0–30 min, verifying the excellent recognition specificity of the system toward γ-HCH and its ability to effectively distinguish HCH isomers. Although polyhydroxy compounds such as glucose can form reversible complexes with boronic acid moieties, their binding affinity is substantially weaker than that of γ-HCH at the optimized pH 6, and they trigger no noticeable fluorescence quenching [51]. Common inorganic ions also cause no significant interference with probe fluorescence.

In this work, we systematically investigated the 15-day long-term storage stability of the as-prepared quantum dot probe at 4 °C, 25 °C and 37 °C (Figure S7C), as well as its anti-photobleaching behavior under continuous excitation illumination for 120 min (Figure S7D), with all fluorescence data normalized for comparative analysis. Storage tests reveal that the fluorescence attenuation rate increases with elevated storage temperature: low-temperature incubation at 4 °C effectively suppresses surface ligand desorption and surface trap formation, yielding optimal storage performance with normalized fluorescence intensity exceeding 96% after 15 days; mild attenuation is observed at 25 °C, suitable for short-term ambient storage; pronounced decay occurs at 37 °C, with normalized intensity falling to 83% after 15 days of incubation. Photostability measurements show the normalized fluorescence intensity remains above 95% within the initial 0–60 min illumination window, indicating outstanding short-term photostability. The decay rate rises moderately after 60 min, and 82% of the initial fluorescence intensity is retained after 120 min continuous irradiation.

The results reveal that the CdSe/ZnS-COOH@3-APBA probe possesses excellent specific recognition and anti-interference performance toward γ-HCH, as well as favorable storage stability and robust anti-photobleaching capability for practical fluorescence detection.

### 2.7. Standard Addition Recovery in Real Samples

Standard addition recovery experiments were performed to evaluate the applicability of the proposed sensing method in real matrices including river water, apple juice and vegetable juice (Table 2). The recoveries at different spiking levels ranged from 98.80% to 101.60%, with relative standard deviations (RSDs) below 5.00% (n = 3 for each spiked level). To further validate the reliability of the developed sensor, parallel detection was conducted using GC-MS as a reference method (Table S5). The recoveries obtained by GC-MS were 99.00–101.75%, and the relative errors between the two methods were less than 3%. The results verify that the proposed method exhibits excellent accuracy and repeatability in complex matrices, and can be reliably applied to the sensitive quantification of γ-HCH residues in practical samples.

### 2.8. Superiority of the Detection Performance

This method was compared with reported enzyme biosensors [9,10], electrochemical sensors [14,52], and SERS detection techniques [13] (Table 3). While some electrochemical and enzyme sensors offer lower LODs, they commonly suffer from enzyme inactivation, complicated electrode fabrication, and narrow linear ranges. Unlike bulky instrumental methods such as GC-MS [12], our fluorescent sensing system requires simple pretreatment and operation at low cost without large precision instruments, making it ideal for on-site rapid screening; moreover, it features favorable stability and strong anti-matrix interference capacity, delivering balanced analytical performance and serving as a reliable novel strategy for sensitive trace quantification of γ-HCH.

### 2.9. Risk Assessment of Cd^2+^ Leaching from the Probe

In a weakly acidic ethanol–water system (volume ratio = 2:3, pH = 6), ethanol only induces mild isotropic etching on the surface of quantum dots, which does not damage the intrinsic core–shell lattice structure or generate penetrating pores within the ZnS encapsulation layer [53]. The low proton activity at this pH slows the hydrolytic corrosion of the ZnS shell. Furthermore, the synergistic effect between ethanol-induced etching, aqueous hydrolysis, and oxidation is weak [54]. Consequently, the quantum dots maintain structural stability during short-term fluorescence detection, and the risk of Cd^2+^ leaching is controllable. pH serves as a critical parameter determining the dissolution extent of the ZnS shell and modulating the rate and cumulative amount of Cd^2+^ release: near-neutral to neutral conditions (pH 5.96–7.4) preserve the structural integrity of the ZnS shell with extremely low Cd^2+^ leakage [55,56], whereas elevated acidity accelerates shell corrosion and facilitates the dissociation of the CdSe core accompanied by Cd^2+^ liberation [57].

Although Cd^2+^ leaching risk is controllable under optimized measurement conditions, the probe still has potential safety hazards. The ZnS encapsulation layer cannot completely inhibit Cd^2+^ effusion in the acidic intracellular microenvironment, which may lead to potential cytotoxicity [58]. Moreover, waste liquid containing quantum dots is categorized as laboratory hazardous waste and must be centrally collected for professional harm-reducing treatment; if discarded materials enter the natural environment, they will continuously erode and release cadmium ions, resulting in secondary heavy-metal pollution in water and soil [59]. Hence, standardized operating procedures and strict waste management protocols should be adopted for the practical application of this probe.

To avoid the safety risks originating from the above-mentioned heavy-metal leaching, our future research will be devoted to developing novel low-toxicity [60] and environmentally friendly sensing materials for the detection of environmental pollutants [61,62].

## 3. Experimental Section

### 3.1. Materials

CdSe/ZnS core–shell quantum dots were purchased from Jiangsu XFNANO Materials Tech Co., Ltd. (Nanjing, China). 3-Aminophenylboronic acid (3-APBA, 98% purity), L-phenylalanine (99% purity), benzoic acid (99.5% purity), 4-mercaptobenzoic acid (90% purity), aniline (99.5% purity), 1-(3-dimethylaminopropyl)-3-ethylcarbodiimide hydrochloride (EDC·HCl, 98% purity), N-hydroxysuccinimide (NHS, 98% purity), four hexachlorocyclohexane isomers (α-HCH, β-HCH, γ-HCH, δ-HCH, ≥99% purity), dichlorodiphenyltrichloroethane (DDT, 98% purity), chlordane (CDN, 98% purity), aldrin (ALD, 98% purity), humic acid (HA), and various inorganic salts including sodium chloride, potassium chloride, calcium chloride and magnesium sulfate were purchased from Aladdin Reagent Co., Ltd. (Shanghai, China). Anhydrous ethanol (99.7% purity) was purchased from Sinopharm Chemical Reagent Co., Ltd. (Beijing, China). Phosphate-buffered saline (PBS, 0.01 M, pH 6) was freshly prepared by mixing aqueous solutions of sodium dihydrogen phosphate dihydrate (NaH_2_PO_4_·2H_2_O) and disodium hydrogen phosphate dodecahydrate (Na_2_HPO_4_·12H_2_O), with pH adjusted to 6 using 0.1 M hydrochloric acid (HCl) or sodium hydroxide (NaOH) aqueous solutions.

### 3.2. Instrumentation

TEM and HRTEM characterizations were performed on a Zeiss Libra 200 microscope (Carl Zeiss, Oberkochen, Germany) to observe the micromorphology, particle size and lattice fringes of quantum dots. UV–vis absorption spectra were recorded using a TU-1900 ultraviolet–visible spectrophotometer (Beijing Purkinje General Instrument Co., Ltd., Beijing, China). SSF spectra and TRFL were collected on an FLS-920 time-resolved fluorescence spectrometer (Edinburgh Instruments, Livingston, UK). FTIR spectra were acquired with a Nicolet iS50 spectrometer (Thermo Fisher Scientific, Waltham, MA, USA). XPS measurements were carried out on a Thermo ESCALAB 250Xi spectrometer (Thermo Fisher Scientific, USA).

### 3.3. Computational Details

First-principle-based calculations were performed within density-functional theory (DFT) as implemented in the Vienna Ab Initio Simulation Package (VASP 5.4.4, Vienna, Austria) [63]. The frozen-core projector augmented-wave (PAW) method [64] was utilized to treat core-valence electron interactions. The exchange-correlation potential was described by the generalized gradient approximation (GGA) with the Perdew–Burke–Ernzerhof (PBE) functional [65]. An energy cutoff of 400 eV was adopted to guarantee full convergence. The Brillouin zone was sampled via a Γ-centered 1 × 1 × 1 k-point mesh. Structural relaxation was carried out using the conjugate gradient (CG) algorithm to simultaneously minimize the total energy and interatomic forces. The convergence criterion for total energy was set to 10^−5^ eV, and the residual force on each atom was converged to 0.05 eV/Å. Furthermore, the DFT-D3 dispersion correction was incorporated to capture van der Waals (vdW) interactions [66].

The 3D structures of 3-APBA and γ-HCH used for molecular docking were downloaded from the PubChem database and subjected to energy minimization under the MMFF94 force field. Molecular docking simulations were performed using AutoDock Vina 1.2.3 [67]. Prior to docking, the processed small-molecule structures were converted into the PDBQT format required by AutoDock Vina 1.2.3 via ADFRsuite 1.03 [68]. The exhaustiveness of the global search was set to 32, while all other parameters were kept as default. The docking conformation with the highest binding score was selected as the optimal binding pose. Finally, PyMOL 2.5.2 was utilized for visual analysis of the docking results.

### 3.4. Preparation of 3-APBA-Modified CdSe/ZnS-COOH Quantum Dots

The 3-APBA functionalized CdSe/ZnS-COOH quantum dots were synthesized following a previously reported procedure [69]. The surface carboxyl groups of CdSe/ZnS-COOH quantum dots were first activated. Specifically, 50 μL of carboxylated CdSe/ZnS-COOH quantum dots (8 μM) were mixed with 15.3 μL EDC (10 mg/mL) and 9 μL NHS (10 mg/mL) in 100 μL PBS (10 mM, pH 6.0), and the mixture was stirred for 30 min. Afterwards, the pH of the reaction solution was adjusted to 8.0 using PBS (10 mM, pH 8.5), followed by the addition of 3-APBA. After gentle stirring for 3 h, the mixture was purified via ultrafiltration using a 30 kDa MWCO membrane to remove excess unreacted 3-APBA. The final product was diluted to 5 mL and stored for further characterizations and subsequent sensing experiments.

Two distinct surface modification strategies were adopted to construct another four modified quantum dot probes. CdSe/ZnS-COOH@Phe and CdSe/ZnS-NH_2_@BA were fabricated via an EDC/NHS-mediated amide coupling reaction [69]. Using bare CdSe/ZnS core–shell quantum dots as starting materials, CdSe/ZnS@4-MBA and CdSe/ZnS@AN were obtained through ligand exchange [70].

### 3.5. Detection of γ-HCH

Typically, 50 μL CdSe/ZnS-COOH@3-APBA dispersion (80 nM), 50 μL 10 mM PBS (pH 6.0) supplemented with 200 mM NaCl, and serial concentrations of γ-HCH (10–180 nM) were sequentially added into a cuvette. The mixture was diluted to a final volume of 5 mL with ultrapure water and incubated at 25 °C for 10 min. Fluorescence spectra were recorded over the range of 400–600 nm with an excitation wavelength of 360 nm; both excitation and emission slit widths were set to 10 nm. To eliminate background matrix interference, the relative fluorescence quenching efficiency (F_0_ − F)/F_0_ was adopted to quantify the sensing signal, where F and F_0_ correspondingly represent the fluorescence intensities recorded with and without γ-HCH, respectively. All fluorescence measurements were carried out in three parallel replicates.

### 3.6. Analysis of Real Samples

For this work, farmland surface water, fresh apples, and vegetable matrices were collected as real samples. For water samples, suspended solids were removed via static sedimentation, followed by vacuum filtration with a 0.22 μm aqueous filter membrane; the resulting filtrate was reserved for subsequent analysis. For fruit and vegetable samples, surface contaminants were wiped off, and the samples were chopped and homogenized prior to constant shaking-assisted extraction with anhydrous ethanol. After centrifugation, the supernatant was collected and filtered through a 0.22 μm organic-solvent-resistant filter membrane. The filtrate was then diluted with an ethanol–water mixture to obtain test solutions.

For GC-MS analysis, blank river water, apple juice and vegetable juice samples were used. A standard target analyte solution was spiked into blank raw matrices, fully mixed and equilibrated to yield samples with matrix-spiked concentrations of 20, 60 and 140 nM. The spiked samples were filtered with a 0.22 μm aqueous mixed cellulose ester syringe filter, the initial 3 mL filtrate was discarded and the subsequent filtrate was retained. n-Hexane–acetone (4:1, *v*/*v*) served as the extraction solvent at a filtrate/solvent volume ratio of 1:2. The mixture was vortexed at 2500 rpm for 5 min for extraction and stood at room temperature for 10 min for phase separation. After centrifugation at 8000 rpm and 25 °C for 10 min, the upper organic supernatant was carefully collected to avoid impurity contamination and serially diluted to constant volume in Class A 10 mL and 25 mL volumetric flasks using n-hexane–acetone (4:1, *v*/*v*) to prepare test solutions for GC-MS measurement.

## 4. Conclusions

Focusing on the practical demand for trace detection of γ-HCH, this work integrates surface functionalization with fluorescence sensing technology, which not only preserves the excellent optical stability of QDs but also endows them with specific recognition capability toward γ-HCH.

When compared with conventional detection methods and similar fluorescent probes, the proposed sensing system has core advantages summarized in three aspects: (i) high detection sensitivity: a favorable linear response is obtained in the range of 10–140 nM (LOD = 2.62 nM, *R*^2^ = 0.9953); (ii) excellent target specificity: precise recognition of γ-HCH is achieved through the synergistic action of multiple interactions and spatial configuration matching, effectively distinguishing γ-HCH from other organochlorine pesticides and interfering substances, while interference from polyhydroxy compounds such as glucose is effectively avoided, endowing the system with outstanding anti-interference performance; and (iii) strong practicability: the optimized reaction conditions are highly compatible with practical detection scenarios, and this method features facile operation and no requirement for large-scale precision instruments, significantly improving detection efficiency compared with traditional techniques such as gas chromatography, with lower cost and wider applicability.

Mechanistic analysis reveals that the formation of non-fluorescent ground-state-associated complexes between γ-HCH and the probe induces the fluorescence quenching response. Meanwhile, the probe can maintain favorable dispersity in detection media without particle aggregation, which effectively eliminates detection deviations arising from aggregation and imparts outstanding detection accuracy and reproducibility to this sensing system.

In summary, the fluorescent sensing system constructed in this work not only overcomes the limitations of traditional γ-HCH detection methods, but also achieves the integration of high sensitivity, specificity, and practicability, providing a novel technical route for the rapid and accurate detection of γ-HCH in environmental and food matrices. The design concept and technical scheme possess promising application potential, and can offer a valuable reference for the detection of analogous organic pollutants.

## Figures and Tables

**Table 1:** Double-exponential kinetic fitting parameters of the fluorescence lifetime of the probe before and after binding with γ-HCH.

Sample	τ_1_ (ns)	f_1_ (%)	τ_2_ (ns)	f_2_ (%)	Average Lifetime τ_avg (ns)
CdSe/ZnS-COOH@3-APBA	1.0993	42.17	3.5757	57.83	1.421
CdSe/ZnS-COOH@3-APBA/γ-HCH	1.0630	40.14	3.5103	59.86	1.435

**Table 2:** Determination of γ-HCH in river water, apple and vegetable samples using a self-fabricated fluorescence sensor.

Samples	Added (nM)	Detected (nM)	Recovery (%)	RSD (%, n = 3)
River water	20	20.32	101.60	0.80
	60	60.18	100.30	0.35
	140	140.09	100.06	0.48
Apple juice	20	19.76	98.80	0.88
	60	60.15	100.25	0.40
	140	140.35	100.25	0.63
Vegetable juice	20	19.84	99.20	0.92
	60	60.52	100.87	0.61
	140	140.48	100.34	0.75

**Table 3:** Comparison of analytical performances of γ-HCH determination methods.

Method	Probe	Linear Range (nM)	Detection Limit (nM)	Reference
Enzyme Biosensor	ZGOM@L	0.172–48.13	0.069	[9]
Biosensor	Polyaniline-Based	6.88 × 10^−3^–1.55 × 10^−1^	6.88 × 10^−3^	[15]
GC–MS	DES@MGO	34.39–343.84	1.032	[10]
GC	ECD	0.344–34.39	0.103	[12]
SERS	4-MPBA-ANHCs	1–100	1.032	[13]
Electrochemistry	MWCNT-MIP	0.1–1 × 10^6^	0.1	[14]
Electrochemistry	PoPD@TiO2 NTs	100–1 × 10^4^	30	[52]
Fluorescence	3APBA-CdSe/ZnS	10–140 nM	2.62	This work

## Data Availability

The original contributions presented in this study are included in the article. The raw data supporting the conclusions of this article will be made available by the authors on request. Inquiries can be directed to the corresponding authors.

## Supplementary Materials

The following supporting information can be downloaded at: https://www.mdpi.com/article/10.3390/molecules31172989/s1, Figure S1. Size distribution histogram of CdSe/ZnS-COOH quantum dots; Figure S2. (A) Excitation and emission (EX/EM) spectra of CdSe/ZnS-COOH quantum dots; (B) excitation and emission (EX/EM) spectra of CdSe/ZnS-COOH@3-APBA probe. Figure S3. High-resolution XPS C 1s and O 1s spectra of CdSe/ZnS-COOH together with their peak-fitting results. High-resolution XPS B 1s, N 1s, C 1s and O 1s spectra of CdSe/ZnS-COOH@3-APBA and their peak-fitting results. Figure S4. (A) Fluorescence emission spectra of CdSe/ZnS-COOH, CdSe/ZnS-COOH@3-APBA, and CdSe/ZnS-COOH@3-APBA with γ-HCH. (B) Comparison of fluorescence quenching efficiency of CdSe/ZnS-COOH modified with different functional groups toward γ-HCH. Figure S5. (A) XPS survey spectrum of CdSe/ZnS-COOH@3-APBA probe after incubation with γ-HCH; (B) high-resolution Cl 2p XPS spectrum. Figure S6. Residual diagnostic plots for the linear calibration model. (A) Residuals versus concentration; (B) Histogram of residuals; (C) Residuals versus predicted response; (D) Normal probability plot of residuals. Figure S7. (A) Molecular docking model of 3-APBA and γ-HCH; the yellow dashed line denotes the halogen bond between boron and chlorine atoms. (B) Time-dependent fluorescence responses of the CdSe/ZnS-COOH@3-APBA probe toward four HCH isomers. (C) Normalized fluorescence intensity changes of the CdSe/ZnS-COOH@3-APBA probe stored at different temperatures over 15 days. (D) Normalized fluorescence intensity variations of the CdSe/ZnS-COOH@3-APBA probe under continuous light irradiation for 120 min. Table S1. XPS atomic percentages of various elements for CdSe/ZnS quantum dots before and after surface modification. Table S2. XPS peak-fitting parameters for C 1s and O 1s spectra of CdSe/ZnS-COOH quantum dots. Table S3. XPS peak fitting parameters for C 1s, N 1s, O 1s and B 1s spectra of CdSe/ZnS COOH@3 APBA. Table S4. Adsorption energies between CdSe/ZnS-COOH@3-APBA probe and four HCH isomers. Table S5. Comparison of detection results of target analytes in real samples between the fluorescent sensing method and GC-MS standard method.

## Abbreviations

The following abbreviations are used in this manuscript:

γ-HCH	γ-hexachlorocyclohexane (Lindane)
QDs	Quantum Dots
3-APBA	3-Aminophenylboronic Acid
HCH	Hexachlorocyclohexane
GC-MS	Gas Chromatography–Mass Spectrometry
SERS	Surface-Enhanced Raman Scattering
PET	Photoinduced Electron Transfer
TEM	Transmission Electron Microscopy
HRTEM	High-Resolution Transmission Electron Microscopy
UV–vis	Ultraviolet–Visible
SSF	Steady-State Fluorescence
TRFL	Time-Resolved Fluorescence Lifetime
FTIR	Fourier Transform Infrared
XPS	X-ray Photoelectron Spectroscopy
DFT	Density-Functional Theory
VASP	Vienna Ab Initio Simulation Package
PAW	Projector Augmented-Wave
GGA	Generalized Gradient Approximation
PBE	Perdew–Burke–Ernzerhof
CG	Conjugate Gradient
^11^B NMR	^11^B Nuclear Magnetic Resonance Spectroscopy
DFT-D3	Dispersion-Corrected DFT-D3
ΔE	Energy Difference
PDOS	Partial Density of States
FRET	Fluorescence Resonance Energy Transfer
IFE	Inner Filter Effect
LOD	Limit of Detection
HPLC	High-Performance Liquid Chromatography
RSDs	Relative Standard Deviations
L-Phe	L-phenylalanine
BA	Benzoic Acid
4-MBA	4-Mercaptobenzoic Acid
AN	Aniline
EDC · HCl	1-Ethyl-3-(3-dimethylaminopropyl) carbodiimide hydrochloride
NHS	N-Hydroxysuccinimide
DDT	Dichlorodiphenyltrichloroethane
CDN	Chlordane
ALD	Aldrin
HA	Humic Acid
NaCl	Sodium Chloride
KCl	Potassium Chloride
CaCl_2_	Calcium Chloride
MgSO_4_	Magnesium Sulfate
PBS	Phosphate-Buffered Saline
NaH_2_PO_4_·2H_2_O	Sodium Dihydrogen Phosphate Dihydrate
Na_2_HPO_4_·12H_2_O	Disodium Hydrogen Phosphate Dodecahydrate
HCl	Hydrochloric Acid
NaOH	Sodium Hydroxide
