# Supplementary material for: Turn-Off Fluorescent Sensor Based on 3-Aminophenylboronic Acid-Modified CdSe/ZnS Quantum Dots for Specific Detection of γ-Hexachlorocyclohexane (Lindane)
Source: Molecules. 2026 Aug 26;31(17):2989. doi: 10.3390/molecules31172989 (PMC13567623; doi:10.3390/molecules31172989)
Supplement: Supplementary file 1 [file molecules-31-02989-s001.zip › molecules-4451552-supplementary.pdf]

Turn-Off Fluorescent Sensor Based on 3-Aminophenylboronic Acid-Modified CdSe/ZnS Quantum Dots for Specific Detection of γ-Hexachlorocyclohexane (Lindane)

Dongdong Shi [1] and Guiqin Yan [2,*]

[1] School of Chemistry and Chemical Engineering, Shanxi Normal University, Taiyuan 030031, China; 118111005@stu.sxnu.edu.cn
[2] School of Life Sciences, Shanxi Normal University, Taiyuan 030031, China
* Correspondence: gqinyan2013@126.com

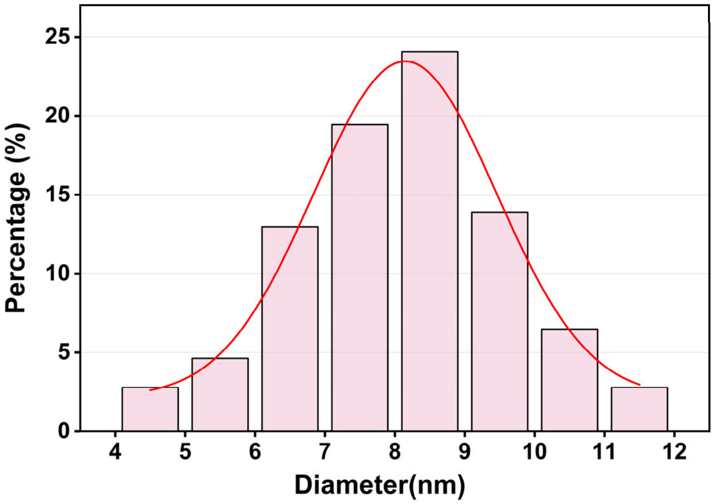

Figure S1. Size distribution histogram of CdSe/ZnS-COOH quantum dots

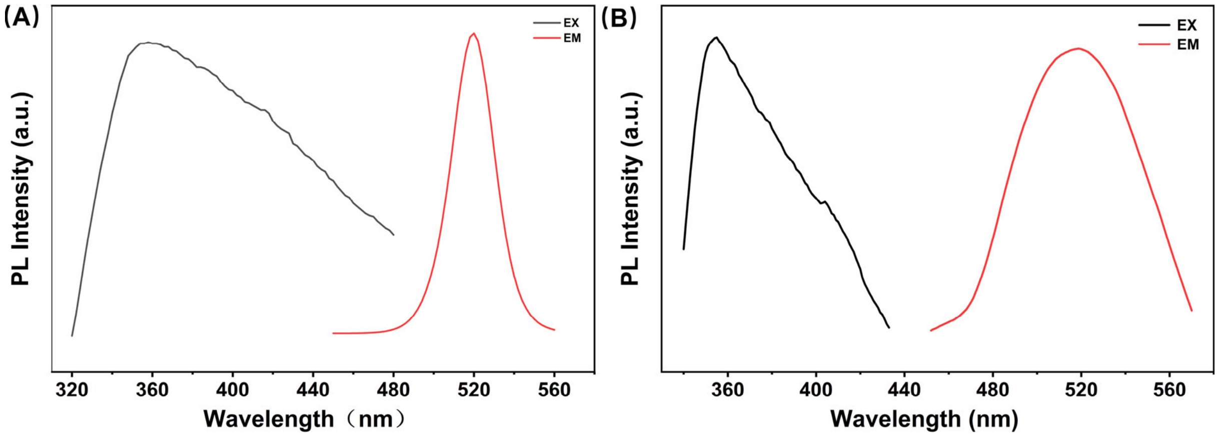

Figure S2. (A) Excitation and emission (EX/EM) spectra of CdSe/ZnS-COOH quantum dots; (B) excitation and emission (EX/EM) spectra of CdSe/ZnS-COOH@3-APBA probe.

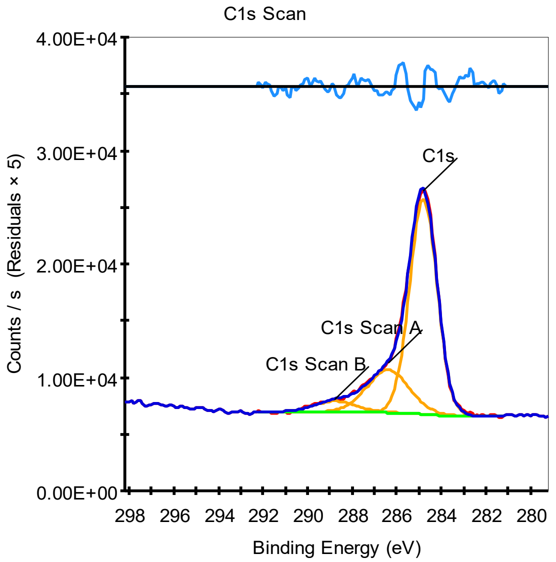
C1s Scan
4.00E+04
3.00E+04
2.00E+04
1.00E+04
0.00E+00
Counts / s (Residuals × 5)
C1s
C1s Scan A
C1s Scan B
298 296 294 292 290 288 286 284 282 280
Binding Energy (eV)

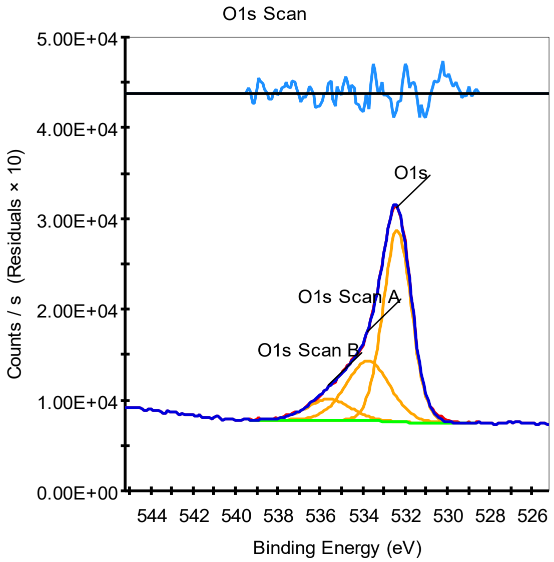
O1s Scan
5.00E+04
4.00E+04
3.00E+04
2.00E+04
1.00E+04
0.00E+00
Counts / s (Residuals × 10)
O1s
O1s Scan A
O1s Scan B
544 542 540 538 536 534 532 530 528 526
Binding Energy (eV)

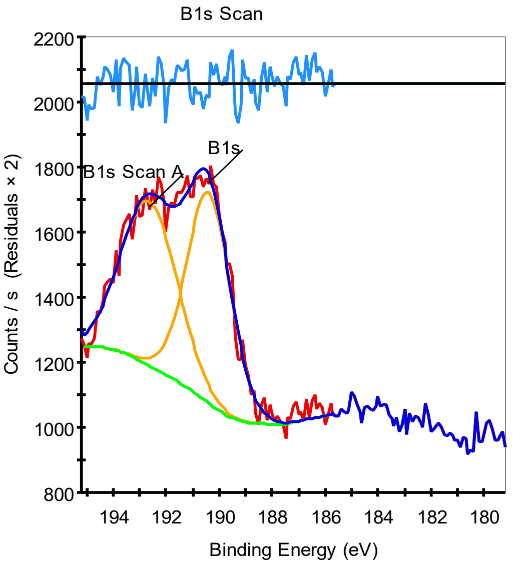
B1s Scan
2200
2000
1800
1600
1400
1200
1000
800
Counts / s (Residuals × 2)
B1s
B1s Scan A
194 192 190 188 186 184 182 180
Binding Energy (eV)

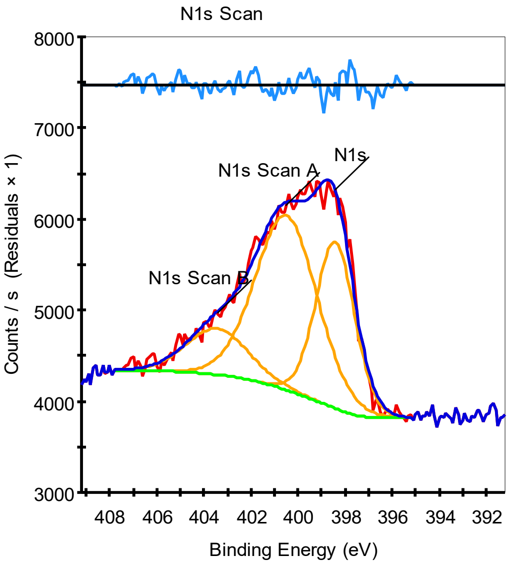
N1s Scan
8000
7000
6000
5000
4000
3000
Counts / s (Residuals × 1)
N1s
N1s Scan A
N1s Scan B
408 406 404 402 400 398 396 394 392
Binding Energy (eV)

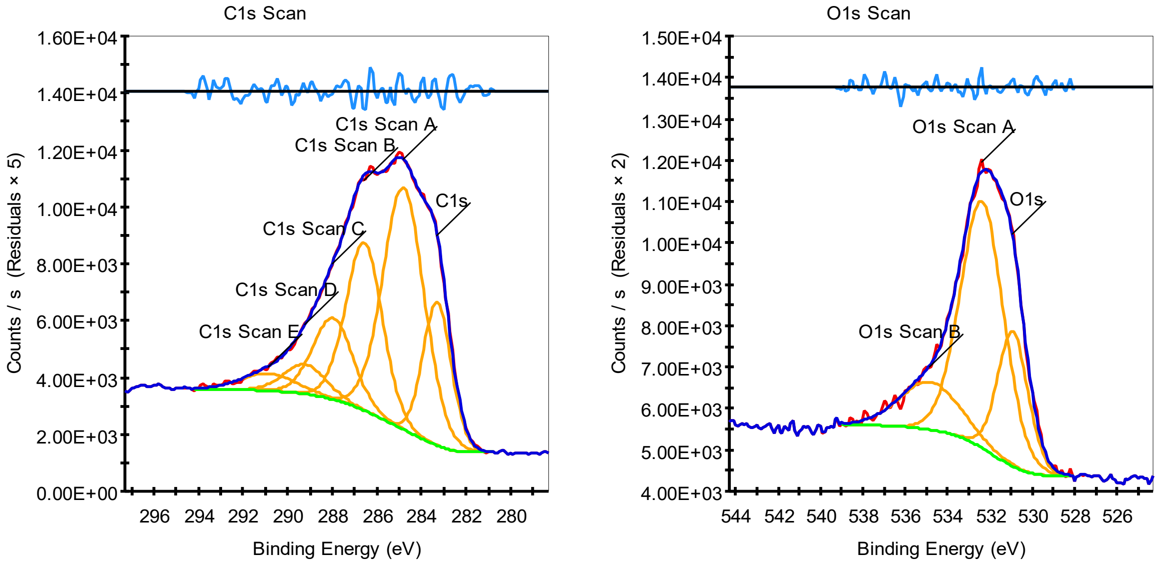

Figure S3. High-resolution XPS C 1s and O 1s spectra of CdSe/ZnS-COOH together with their peak-fitting results. High-resolution XPS B 1s, N 1s, C 1s and O 1s spectra of CdSe/ZnS-COOH@3-APBA and their peak-fitting results. The blue curve denotes the raw experimental spectrum; orange curves are the deconvoluted sub-peaks; the red envelope is the overall fitted spectrum, which matches the raw data. The green curve represents the subtracted fitted background baseline, and the black horizontal line corresponds to the fitting residual curve.

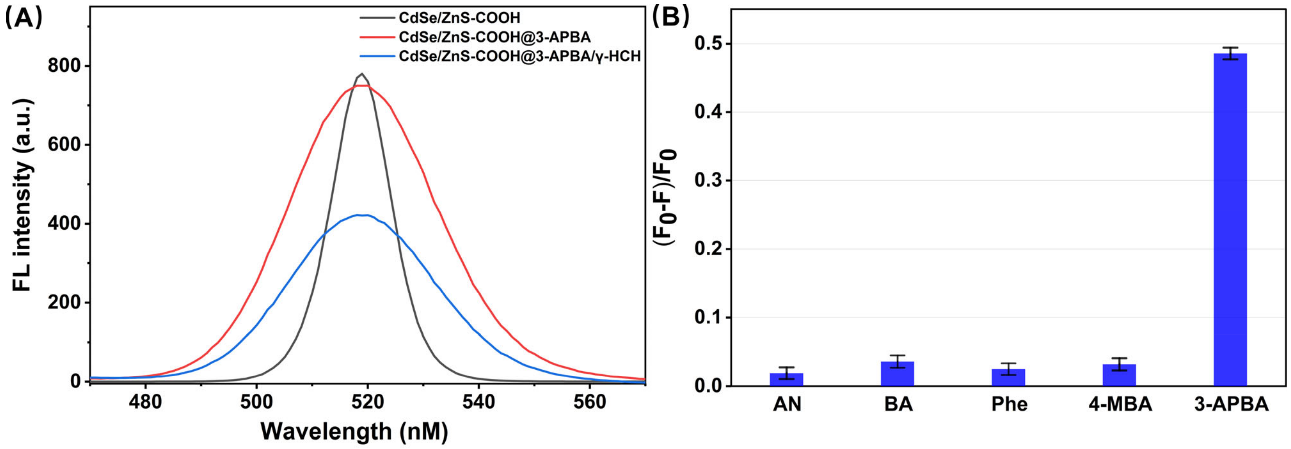

Figure S4. (A) Fluorescence emission spectra of CdSe/ZnS-COOH, CdSe/ZnS-COOH@3-APBA, and CdSe/ZnS-COOH@3-APBA with γ-HCH. (B) Comparison of fluorescence quenching efficiency of CdSe/ZnS-COOH modified with different functional groups toward γ-HCH.

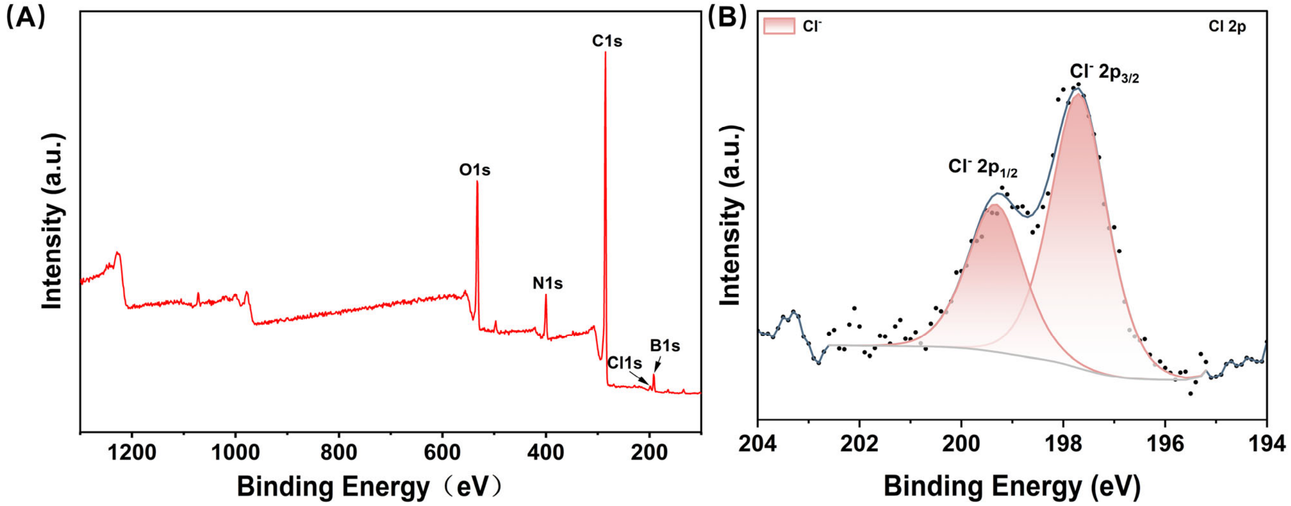

Figure S5. (A) XPS survey spectrum of CdSe/ZnS-COOH@3-APBA probe after incubation with γ-HCH; (B) high-resolution Cl 2p XPS spectrum.

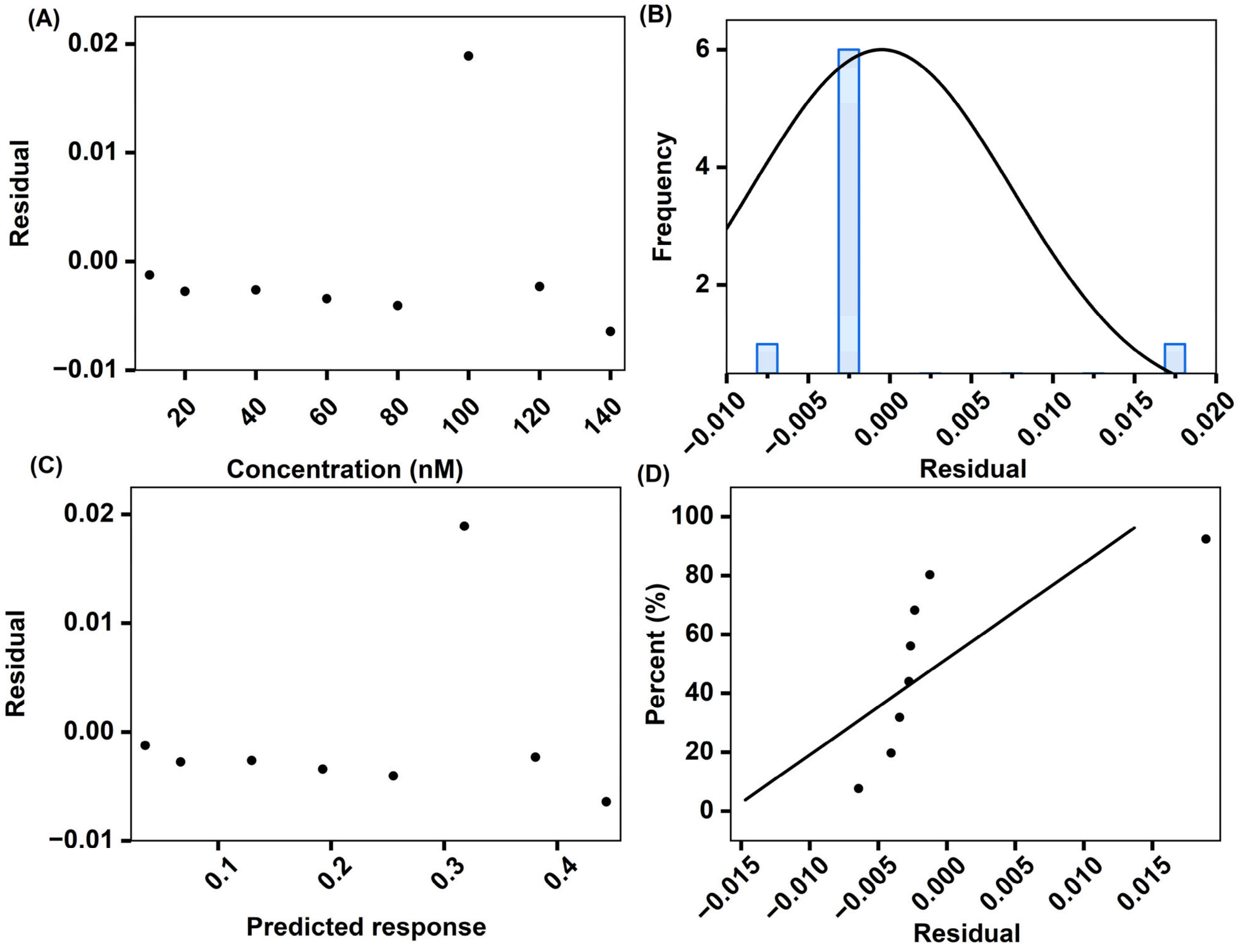

Figure S6. Residual diagnostic plots for the linear calibration model. (A) Residuals versus concentration; (B) Histogram of residuals; (C) Residuals versus predicted response; (D) Normal probability plot of residuals.

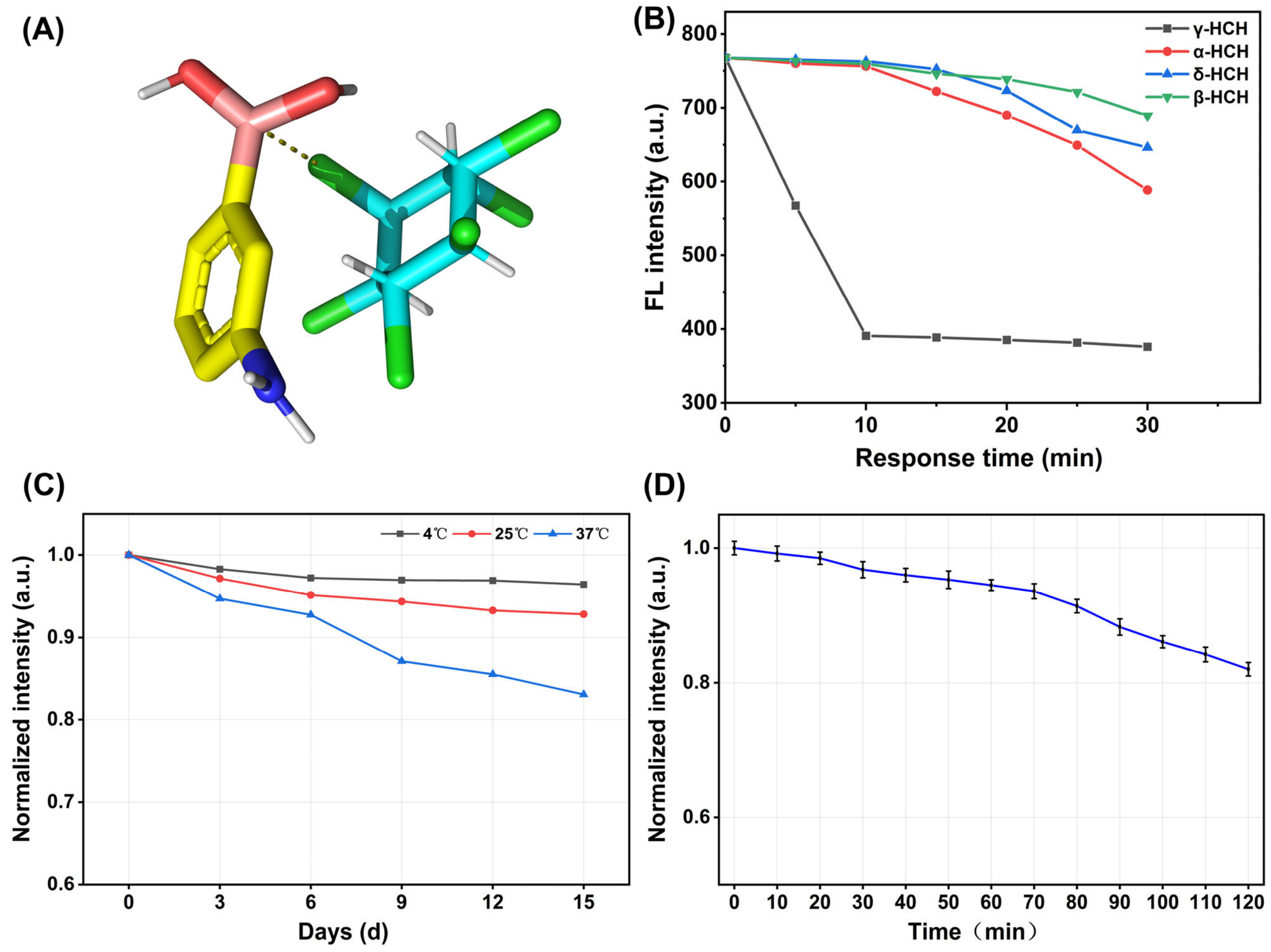

Figure S7. (A) Molecular docking model of 3-APBA and γ-HCH; the yellow dashed line denotes the halogen bond between boron and chlorine atoms. (B) Time-dependent fluorescence responses of the CdSe/ZnS-COOH@3-APBA probe toward four HCH isomers. (C) Normalized fluorescence intensity changes of the CdSe/ZnS-COOH@3-APBA probe stored at different temperatures over 15 days. (D) Normalized fluorescence intensity variations of the CdSe/ZnS-COOH@3-APBA probe under continuous light irradiation for 120 min.

Table S1. XPS atomic percentages of various elements for CdSe/ZnS quantum dots before and after surface modification

Element	Atomic (%) (unmodified)	Atomic (%) (modified)	Variation description
Se 3d	0.53 %	0.08 %	The quantum dot surface is covered by organic ligands, which attenuate the photoelectron signal of surface-located Se species and reduce its relative atomic concentration.
Cd 3d	0.25 %	0.06 %	The overlying organic coating screens the photoelectron emission from the Cd-containing core domain, leading to a decline in the measured Cd atomic fraction.
S 2p	5.06 %	1.22 %	Surface organic overlayer suppresses the photoelectron intensity originating from the inner-core S component.
Zn 2p	0.79 %	0.19 %	The outer organic-ligand overlayer attenuates the detectable Zn-related

			photoelectron signal.
B 1s	0 %	8.35 %	Bare quantum dots contain no boron; the appearance of substantial B 1s signal verifies successful grafting of boronic-acid-containing moieties, providing key quantitative evidence for surface modification.
N 1s	0 %	9.60 %	Detected nitrogen originates from 3-APBA. The N 1s signal serves as complementary corroborative evidence together with the B 1s signal.
C 1s	61.01 %	67.19 %	Elevation of carbon atomic fraction arises from the introduction of benzene-ring-bearing organic fragments.
O 1s	32.37 %	13.30 %	Condensation occurs between native surface -COOH groups and amino moieties, resulting in a pronounced reduction in the contribution from free carboxyl-group-related oxygen species.

Table S2. XPS peak-fitting parameters for C 1s and O 1s spectra of CdSe/ZnS-COOH quantum dots

Sample	Binding energy (eV)	FWHM (eV)	Area (cps, eV)	Content (%)
**C 1s**				
C-C/C-H	284.80	1.44	29134.37	72.04%
C-O	286.39	2.21	8996.38	22.25%
C=O	288.77	2.21	2306.87	5.71%
C-C/C-H	284.80	1.44	29134.37	72.04%
**O 1s**				
C=O	532.36	1.57	36003.47	60.85%
C-O	533.74	2.4	17193.81	29.08%
H_2O	535.58	2.4	5951.30	10.07%

Table S3. XPS peak-fitting parameters for C 1s, N 1s, O 1s and B 1s spectra of CdSe/ZnS-COOH@3-APBA

Sample	Binding energy (eV)	FWHM (eV)	Area (cps, eV)	Content (%)
**B 1s**				
B-OH	190.39	1.94	1358.11	50.33%
B-O-C	192.64	2.51	1339.55	49.67%
**N 1s**				
Ar-NH_2	398.41	1.92	3817.09	32.04%
N-(C=O)-	400.49	3.08	6401.20	53.77%
N-O	403.43	3.08	1687.18	14.19%
**C 1s**				
C=C	283.29	1.49	8030.32	15.66%
C-C/C-H	284.80	2.17	20033.70	39.09%
C-O/ C-N	286.57	1.92	12289.57	24.00%
C=O	287.97	2.09	6486.18	12.67%
-COOH	289.23	2.21	2489.04	4.86%
π-π* Sat.	290.86	2.88	1899.33	3.71%
**O 1s**				
C=O	530.91	1.68	5928.01	23.10%
C-O	532.36	2.40	15522.86	60.53%
H_2O	534.82	3.37	4191.75	16.36%

Table S4. Adsorption energies between CdSe/ZnS-COOH@3-APBA probe and four HCH isomers

Probe	Target	Adsorption energy (eV)
CdSe/ZnS-COOH@3-APBA	γ-HCH	-1.165
CdSe/ZnS-COOH@3-APBA	α-HCH	-0.600

CdSe/ZnS-COOH@3-APBA	σ-HCH	-0.474
CdSe/ZnS-COOH@3-APBA	β-HCH	-0.236

(The larger the absolute value of adsorption energy, the stronger the adsorption binding interaction between the probe and the target analyte.)

Table S5. Comparison of detection results of target analytes in real samples between the fluorescent sensing method and GC-MS standard method

**Samples**	**Added (nM)**	**This work**			**GC-MS**			**Relative error (%)**
		**Detected (nM)**	**Recovery (%)**	**RSD (%)**	**Detected (nM)**	**Recovery (%)**	**RSD (%)**	
River water	20	20.32	101.6	0.80	19.95	99.75	1.85	1.85
	60	60.18	100.3	0.35	60.65	101.08	1.20	−0.78
	140	140.09	100.06	0.88	139.1	99.00	1.05	0.71
Apple juice	20	19.76	98.80	0.88	20.28	101.4	2.10	−2.56
	60	60.15	100.25	0.62	59.72	99.53	1.55	0.72
	140	140.35	100.25	0.76	139.4	99.57	1.30	0.68
Vegetable juice	20	19.84	99.20	0.92	20.35	101.75	2.30	−2.51
	60	60.52	100.87	0.48	60.98	101.63	1.75	−0.75
	140	140.48	100.34	0.68	141.7	100.07	1.40	-0.86
